# An Epistemology on the Nature of Polymers

**DOI:** 10.1371/journal.pone.0109403

**Published:** 2014-10-20

**Authors:** Mortéza Laridjani, Pierre Leboucher

**Affiliations:** 1 Laboratoire de Physique des Solides, Université de Paris-Sud, Orsay, France; 2 CNRS – UMR7225, Paris, France; Washington State University, United States of America

## Abstract

Liquids have neither a periodic structure nor the completely random character of gases therefore the detailed study of their x-ray scattering diagram encounters many difficulties. The idea of periodic regularity in molecules of liquid polymers has been an attractive proposition for the simple interpretation of liquid polymer x-ray diagrams. The categorisation of polymer substances as being between a crystal phase with a perfect order and an amorphous disordered state is an over simplification of the complex reality. For obtaining structural information, during the early stages of the application of x-ray diffraction, a near crystalline model of the molecular arrangements in liquids was utilised. However, the results from these investigations led to just an approximation of the crystalline state. Our studies have analysed the real image of Fourier space of liquid polymers, for the first time, using anomalous diffractometry. The findings show the precise atomic structure of liquid polymers when transformed, by cooling, to solid polymers. We demonstrate that there is an intermediate ordered structure, characterised by the real full image of Fourier space. This prominent state of matter, an intermediate ordered structure, is defined by a regular unit cell with a five-fold symmetry. These structural atomic studies contribute to a more detailed understanding of the properties of polymers than the traditional studies of the degree of crystallinity.

## Introduction

### « La science se construit à partir des ruptures épistemologiques successives » Gaston Bachelard-1884-1962

The atomic arrangements of liquid structures, amongst all the various structures of matter, are still the least understood. However, it is well known that liquids may be transferred into solids by a first order discontinuous transformation. By this transition of a liquid to the solid state, solids are divided into two classes: crystalline structures or super-cooled liquids.

Crystalline structures are produced at a sharp freezing point T = T_f_ by forming nuclei with a regular geometry, that grow to become the crystalline solid structure. The Fourier space image of these solid substances consists only of sharp spots or sharp lines that indicate the existence of a long-range order in the atomic network of these substances.

For super-cooled liquids, such as glycerine, as the temperature decreases the liquid will freeze at a glass temperature (Tg) [1] to become an amorphous solid. In these cases long-range order is not present in the amorphous state atomic network and the temperature of the glass transition, Tg, varies with the cooling rate. The Fourier space image of these disordered solids consists only of large coherent diffuse scattering that appears very similar to the x-ray diffraction diagrams of liquids. However, the Fourier space images of these amorphous states have no connection with any crystalline state but are those of a succession of different thermodynamic states with continuously decreasing temperature, as this type of liquid is resistant to the formation of crystal nuclei.

To explain the resistance of such liquid to crystal growth, Frank proposed [2], in atomic terms, the formation of *irregular nuclei* which later on led Bernal to develop the importance of ‘*geometrical factor*’ for the structural determination of liquid states [3]. The behaviour of such liquids was obtained from small molecules such as metallic alloys and it was extrapolated to “synthetic plastics”. Despite knowing that polymers do not form from the gaseous phase and that their liquids do not behave as silver or simple liquids such as water or liquid SiO_2_. Furthermore, polymolecules in the liquid form are very colloidal and require a certain force to come together.

Solid polymers are obtained by polymerisation reactions and not by the solidification of liquid polymers therefore the Gibbs phase rule cannot be applied to them. When solid polymers are heated they melt which indicates that there is a combination of viscous and elastic behaviour. For example, liquid rubber does not transform to a solid state or form from a gaseous state but to obtain a solid polymer from rubber a chemical solution needs to be added (vulcanisation) so that liquid rubber is transformed to elastic solid rubber. In this type of polymer synthesis cross-linking is a requirement and a phase transition does not just occur.

The Fourier space images of solid polymers have two main features: sharp lines superimposed on coherent diffuse halos. The mixed character of these images indicates the complex nature of such compounds. Regardless of this complexity, the nature of synthetic polymers was defined by a two-phase model or fringed micelle theory, by using a rough analogy from crystalline small molecule x-ray diffraction patterns and synthetic polymers with mixed x-ray diagrams.

The categorisation of polymer substances, between a crystal phase with a perfect order (with unit cell structure), and an amorphous disordered state, is an over simplification of the complex reality. Polymer scientists started to study the nature of polymers by applying classical physics to obtain some novel structural identity for these new materials. At the time the methodology for obtaining a real image of Fourier space of the sample was not available by classical diffractometry techniques so only approximations were used. With the development of the theory and application of a novel diffraction technique [4], known as anomalous diffractometry, the real image of Fourier space could be obtained. Using this methodology it has been possible to resolve the subtle atomic structural details.

It is of note that completely amorphous or crystalline structures are rarely encountered. For example it has been shown that the image of Fourier space of washed emeraldine base (EB) by N-methylpyrolidinone (NMP) or tetrahydofuran (THF) produces two different images. For the sample washed by NMP the Fourier space image consisted only of diffuse coherent halos that was consistent with an amorphous polymer. Whereas the image of Fourier space of the sample washed by THF consisted of a limited number of lines superimposed on the coherent diffuse halos. This x-ray diagram was numerically resolved by a new method involving the separation of the lines from the coherent diffuse halos. The comparison of the interference function of the coherent background with other data obtained from washed emeraldine base with NMP (pure amorphous) allowed us to define a reference state of randomness [5].

To show the other extreme, i.e. the crystalline state, we chose bulk polyethylene (CH_2_-CH_2_)_n_, since this substance is recognised as an archetypical crystalline state having an image in Fourier space consisting of selective lines superimposed on the coherent diffuse background [6]. We performed structural studies of low-density polyethylene by the anomalous diffractometry and the results indicated a predominance of local geometry order determination in the atomic network of bulk polyethylene compared to the lattice structure identification [6]. We illustrated the geometry of local order and chemical ordering. Pauling also predicted this chemical ordering by taking into consideration the effect of bond order and resonance in the atomic network of substances.

We deduced that there were two different bond lengths; a single bond between carbon atoms, (C-C), and a double bond between carbon atoms; (C = C) which formed two random networks with different geometrical factors. These networks consist of the small heaps of tetrahedral configurations of carbon atoms in space. We showed experimentally that the structural duality (the crystal chain and atomic close packing model) of diamond could not be adapted for bulk polyethylene [6] and that the atoms in the core of bulk polyethylene were not distributed in a disordered manner. Hence we concluded that the atomic arrangement of solid bulk polyethylene could not be periodic. This result suggested that the traditional concept of a two-phase model and the analogy with diamond structure was an over simplification. Thus, we considered the bulk polyethylene as an intermediary state of matter [6].

This original work proceeded to investigate precisely the full image of Fourier space of liquid polymers in order to understand the transition of liquid state to the solid polymer at the atomic level by studying polyethers. The general formula of a polyether is expressed as [(CH_2_)_m_-O-]_n_. When m = **∞**, the general formula of polyethylene is (CH_2_)_n_; for m = 2 we obtain polyethylene oxide with the hydroxyl groups at both ends, referred to as poly[ethylene glycol] or PEG with a general formula expressed as HO-[CH_2_-CH_2_-O]n-H. For n = 4, 6 and 12, (PEG)n is in liquid form but when n = 20 (molecular weight M = 898–1000) it becomes a solid. This versatility of the physical state of PEG, just by changing the degree of polymerisation (n), makes it ideal for fundamental investigation to understand, at the atomic level, the phase transition of the liquid to solid state in polymers. Anomalous diffractometry permits us to acquire a better understanding of polymers leading to a correct characterisation of their structure.

Using PEG it was possible to obtain a polymer alloy or polymer blend. For instance, by using mixtures of PEG with low molecular masses [M = 200, 300, 600] and tetraethoxysilane it was possible to obtain organic-inorganic nano-composites by the sol-gel process. When these materials were obtained under acidic conditions the PEG were linked to the amorphous SiO_2_ grains and formed an ideal composite with very high transparencies. These new materials have been used for production of glass [7].

In this study we have analysed the full real image of Fourier space of these liquid polymers, for the first time, using anomalous diffractometery. Our findings reveal the precise atomic structures of liquid polymers when transformed, by cooling, to solid polymers.

## Materials and Methods

### Sample Preparation

The solid and liquid state of poly [*ethylene glycol*] PEGs were examined using anomalous diffractometry, at room temperature. Two types of sample holders were used:

- i- a sample holder made from a thin beryllium foil in the form of a rectangular receptacle (cell) with dimensions of φ = 40×20×1.5 mm. This sample holder was used for liquid PEG where M = 200, 300, 600 or 1000.
- ii- a sample holder made of an aluminium based alloy in the form of a frame with the same dimension as the beryllium sample holder. This was used for solid samples of (PEG)_1000_ and the solidified liquid PEG which was obtained by two different methods. The liquid sample was kept in the fridge, (0°C) and once solidified it was quenched in liquid nitrogen. The liquid polymers were kept in the first type of cell (in beryllium) and were cooled *in situ*.

For these experiments, a special low temperature chamber was adapted to the goniometer to avoid undesirable diffuse scattering. This is one of the advantages of anomalous diffractometery, which has parallel incident beams compared to classical diffractometers, which have a focussing circle.

The chamber was cooled down from room temperature (T = 273 K) to the T = 224 K by a free stream of liquid nitrogen inside the chamber. The temperature was controlled automatically by a thermocouple situated near the specimen to regulate the liquid nitrogen flow. The flexibility of goniometer permitted both the incident and scattered beam from the sample to cross through the vacuum path.

## Methods

### Anomalous diffractometer- a new prototype for detecting, at the atomic level, the local geometry of polymers

As liquids have neither a periodic structure nor a completely random gaseous characteristic the detailed study of their diffraction by x-ray presents formidable difficulties, especially in the case of polymers with light components such as C, O or N. The scattering beam from such samples is not intense and is mixed with non-coherent Compton radiation. Hence the type of scattered beam from liquid-state of polymers requires a high performance diffractometer. The aim of this work was to analyse the real image of Fourier space of liquid polymers and eventually studying how liquid polymers, by cooling them, transformed to solid polymers. Obtaining precise data was essential for this study thus we used the anomalous diffractometer.

#### Description of the Anomalous diffractometer

**Figure 1:**
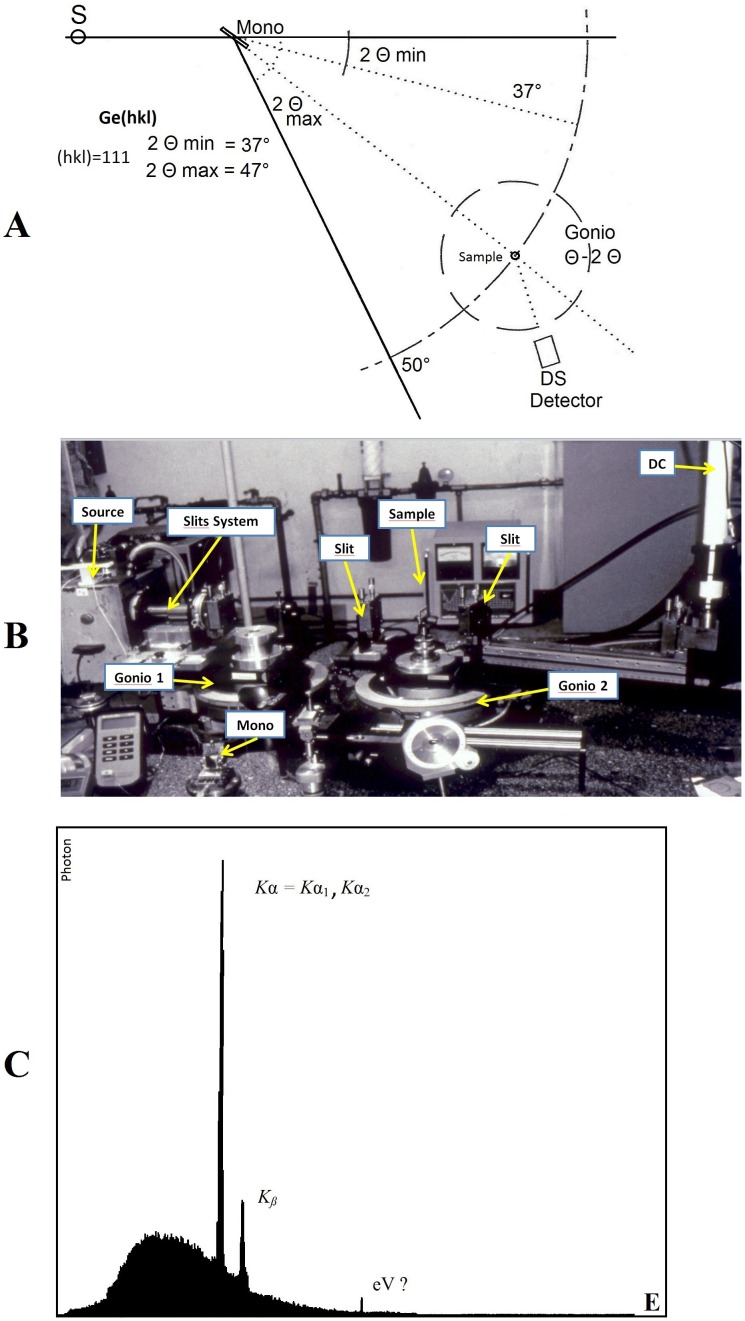
The Original scheme of the Anomalous diffractometer (A). **B-** The anomalous diffractometer consists of three main parts: two goniometers, energy calibrator, data acquisition and processing software. In this complex, the geometrical condition and the optical arrangement was optimised to address any specific question. **C-** The spectrum of direct beam of rotating Ag anode at 50 KV. This was obtained from a prototype solid-state detector. This image shows the continuous and characteristic radiations of Kα (kα1, kα2).

The anomalous diffractometer was designed and built by M.L. It uses a high intensity parallel beam and the instrument has three main parts: the goniometer, an energy window for energy calibration and a data acquisition system with processing software (Figure 1A).

**Figure 2:**
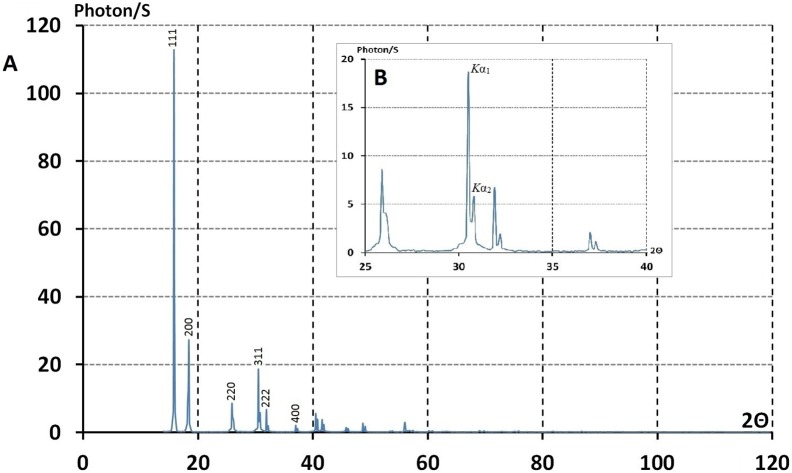
The full real image of Fourier space of polycrystalline, Ni, obtained by Anomalous diffractometer (The Powder method). This image consists only of the sharp diffraction lines; Delta function: The total absence of diffuse scattering indicates a perfect matching of diffraction experiments with the mathematical model, proposed for the atomic arrangement of crystalline substances in periodic space (extinguishment of any diffuse scattering).

**Figure 3:**
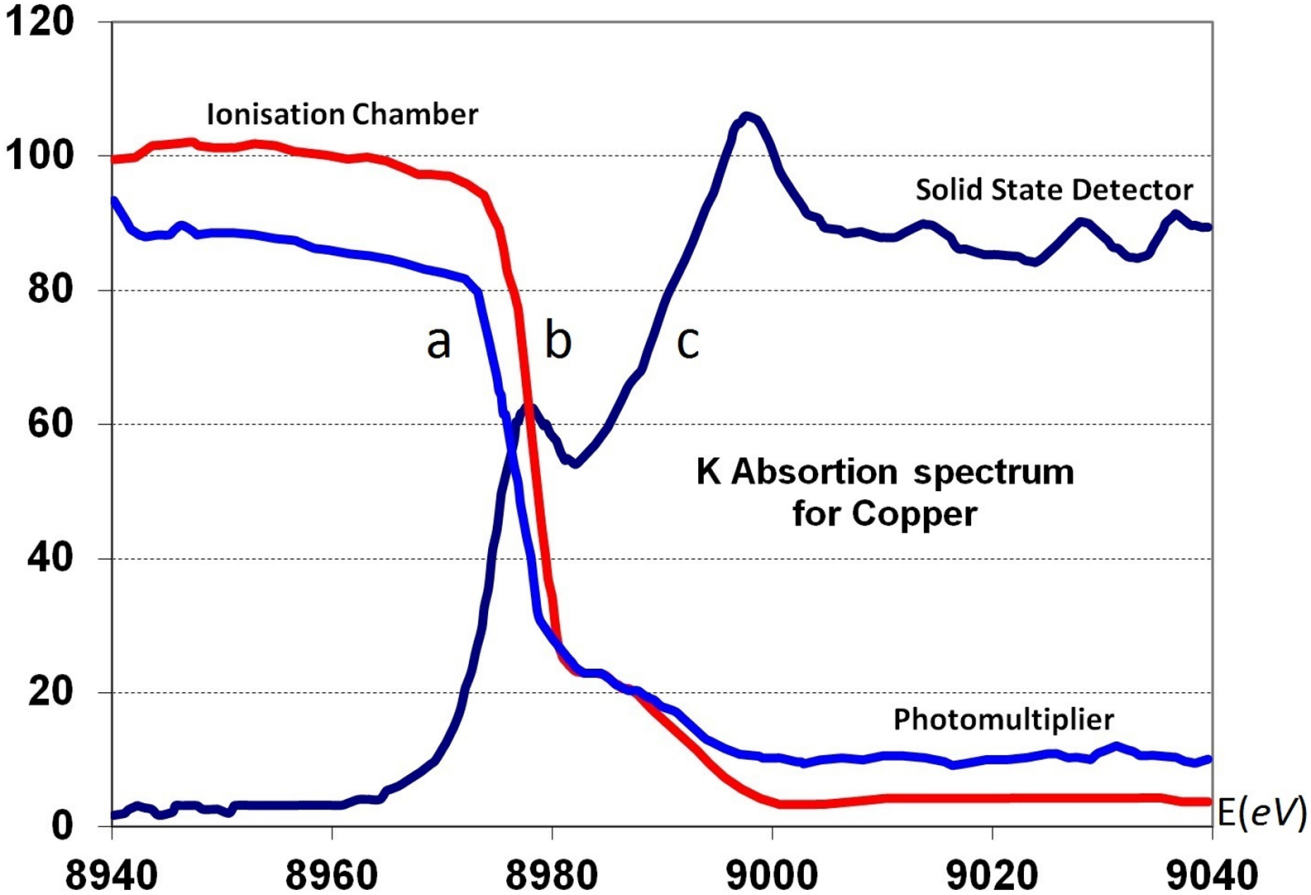
The precise determination of energy by the anomalous diffractometry method. The inflection point in the Fig. (3C) clearly defined the K absorption edge of the element copper. The choice of wavelength (energy) depended on the particular problem. i.e structural studies by the powder method. The real full image of Fourier space was an absolute requirement. λKα was to be far from the K absorption edge of the elements in the sample. Here it was possible to produce a real full image free of fluorescent radiation.

- *a- The goniometer:* the prototype consisted of two goniometers installed on a rotating anode generator (Ag or Cu target tube) (Figure 1B). They were adapted to a parallel beam, which was obtained by a special optical system proposed for small angle scattering technique [8]. Hence, the direct beam, at very small angles, did not over shadow the scattering from the sample. This arrangement also offers the possibility of simultaneously studying very small scattering angles in the same x-ray diffraction diagram, i.e. a full image of Fourier space. The goniometer was used at θ-2θ, θ-θ and 2θ-θ, scanning both in transmission and reflection geometry mode with a resolution of 0.001° degrees. The optical system and geometrical flexibility of the goniometer was finely optimised, to reduce the deviation of observed diffraction lines from the ideal profile (the Delta function) without introducing any background, in the case of ideal crystalline material Figure 2.
- ***b- The energy window for energy calibration:*** The data were acquired by processing software with a prototype of lithium-drifted silicon (Li-Si) solid-state detector (500 g in weight). The detector was connected to a preamplifier and pulse processor.
- ***c- Data acquisition system:*** The pulse processor is a sophisticated signal-processing unit, which provides linear amplification, noise filtering, pulse-pile-up rejection and lifetime correction. The raw pulses are collected by a multi-channel analyser interfaced to a computer [9]. The combination of these functions with high resolution detectors of 150 eV at 8 KeV and 230 eV at 20 KeV is an essential prerequisite for achieving a precise energy window. Depending on the information required from the samples the geometrical condition and optical arrangements of the diffractometer were optimised. Two modes of diffractometry, dispersive energy and non-dispersive energy were performed with this type of energy window.
- ***i- Dispersive energy or variable λ:*** In the dispersive mode a polychromatic component of x-ray radiation from the target was used (Figure 1C) and the energy window was fully open where the energy was variable and θ-2θ was fixed. Large quantities of photons were received by the samples and thus the exposure times were considerably reduced (order of seconds). The brief exposure time was essential for monitoring transitions such as rapid transformations in biomaterials, metastable phases or sample changes under high pressure. The resolution in this dispersive mode was poor since the shadow of the white spectrum was superimposed on the diffraction patterns from the sample. Hence the real Fourier space of the sample was obtained without the direct beam’s shadow.
- ***ii- Non-dispersive energy with variable θ-2θ or fixed λ:*** In this non-dispersive mode the λ energy was selected by two procedures. The first one was to use a flat monochromator (Si, Ge, C, LiF) to calibrate the wavelength of the incident beam providing rapid tunability over the white spectrum of the generator’s target (Figure 1C). The energy calibration was achieved by step scanning across the absorption edge of the heavy elements in the specimen Figure 3. The x-ray diagrams of the specimen obtained from different energies; one near the absorption edge and the other far away, provided the necessary contrast for the Fourier space image. Anomalous x-ray diffraction takes advantage of the dependence of the total atomic scattering f_tot_ (K, E) on the energy (λ) of the incident beam, written as: 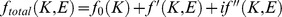 (Figure 3).

This dependence on the energy was sufficient to change the contrast between the two images of the different components of the sample. When the energy (λ) of the incident beam was much higher than the absorption edge, 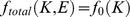 but when the *E* of the incident beam was near the absorption edge of one of the components of the sample the anomalous dispersion effect intervened. This technique allowed us to obtain partial interference and partial atomic pair distribution functions, which gave the precise atomic structural information about the environment of each chemical component in non-crystalline and crystalline materials [4], [10].

The second procedure relied on the fact that in the non-dispersive mode the energy window could be taken as a monochromator in the path of the scattering beam. This was scattered from the sample when irradiated by the monochromatic component (Kα and Kβ) of the target spectrum and any energy of the white spectrum could be used simultaneously (Figure 1C). Therefore in the same experiment, with the same sample, different images of Fourier space with various wavelengths were obtained.

A menu driven software system ran the auto-alignment of the goniometer for each selected wavelength (λ). The correct alignment of the goniometer (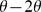) with a precise zero point permitted us to obtain the maximum intensity and a precise scattering angle. The straightforward selection of wavelengths made it feasible to obtain in the same (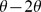) scan a small angle scattering diagram (K≤0.01 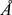 ^−1^). By using the white spectrum where (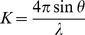), it permitted us to extend K space from 25 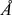 ^−1^ to 30–40 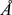 ^−1^.

The anomalous diffractometer was adapted to obtain parallel beams from high intensity sources, which facilitated the elimination of irrelevant scattering such as incoherent background, Compton and fluorescence radiation, from the coherent scattering. These unique properties resulted in an unprecedented quality of Fourier space image of polymers.

### Analysis of Fourier space image of liquid and solid state of PEG

#### The separation procedure of lines from the broad halos

An essential requirement for obtaining a Fourier-space image from solid PEG was the precise separation of selective lines from the coherent diffuse scattering. We neglected the contribution of thermal diffuse scattering (TDS) in the coherent diffuse scattering [11]. In fact the major contribution of TDS was caused by inelastic phonon scattering, arising from acoustic modes in the very close vicinity of the Bragg’s reflection in the crystalline sample. However, with our experimental resolution, it was confined inside the selective lines. The nature of diffuse scattering of solid PEG diagrams was previously studied [4], [6], [30]. In this present work we used the same methodology i.e. the Fourier space image was numerically resolved into coherent diffuse scattering curves and the selective lines. The law of conservation of intensity of the diffracted beam was applied.

The intensity, I (2θ) at each point of the reciprocal space (Fourier space) is the sum of the intensities scattered by two parts: the diffuse coherent scattering (Ic) and selective lines or discrete lines (Is), thus:


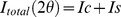
 as the intensity of each point is independent of the mutual arrangement of atoms in the sample’s atomic network.

The Matlab (toolbox of 3860) [5] program was expanded for these analytical separation procedures. Briefly, these operations consisted of selecting a real point of the coherent diffuse scattering curves, (halos curve), Ic (2θ), by comparing its magnitude with the mean intensity (I) of the previous points. Therefore, the missing part of coherent diffuse scattering curve was constructed point by point. With this method of separation procedure two sets of spectra were obtained; one a discrete line spectra and the other a coherent diffuse scattering.

**Figure 4:**
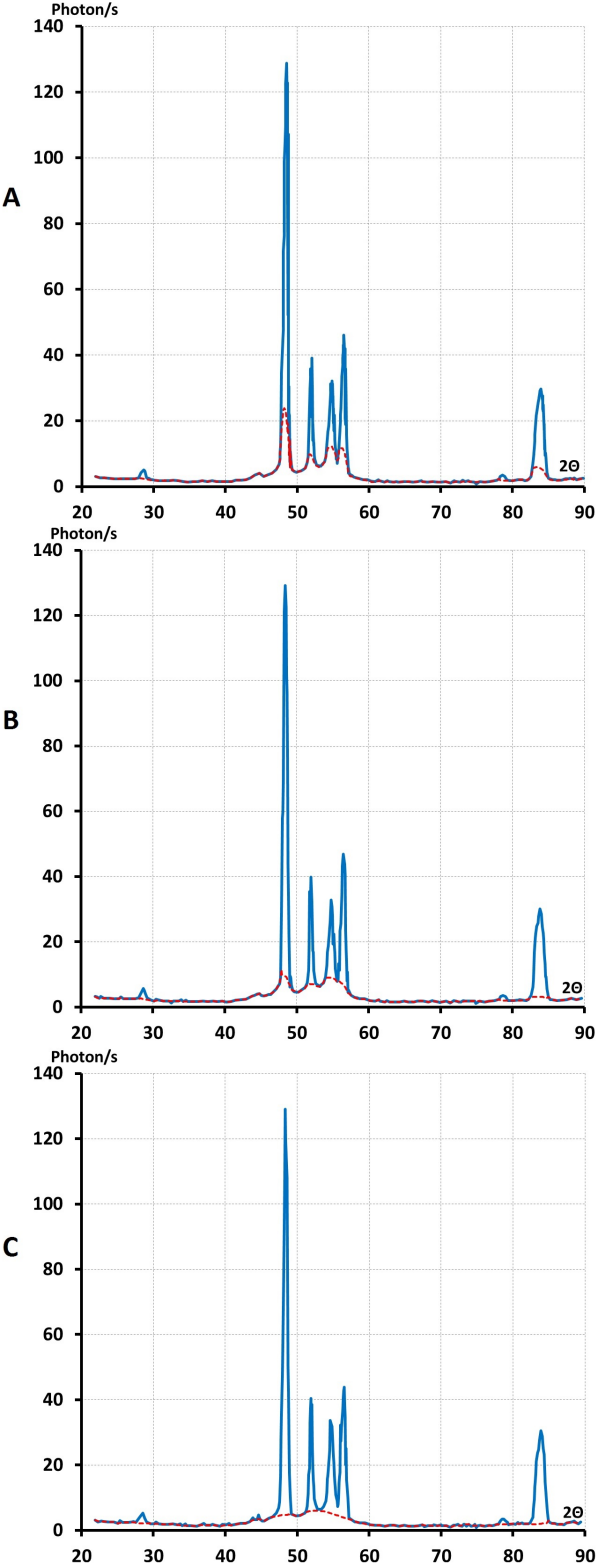
The different stages of analytical separation procedure for obtaining line spectra and coherent diffuse scattering from samples with intermediary structure.

Figure 4 indicates the different stages of the analytical procedure (Figures 4A–C) for obtaining both line spectra and coherent diffuse scattering diagrams. The resolution of the line spectrum and profile of the diffuse scattering curve obtained by this program was linked to the number of times the operation was performed, and the resolution of the steps of (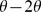) scans for computational averaging. The absence of parasitic diffuse scattering, the facility of eliminating Compton radiation and fluorescence radiation in (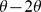) scans, was facilitated by this diffractometer. Without this separation procedure, the information on the local order geometry of atoms in the atomic network of polymers could not be obtained.

### The radial distribution methods

#### The interpretation of Fourier space image of liquid and solid PEG

When applying the method of radial distribution function it was not necessary to make any specific assumption about the sample’s chemical structure. The important requirement was that the experimental x- ray diagrams were to correspond to *a full real image of Fourier space with a very large value* of 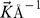 (the scattering vector). This condition was imperative since all interference effects from atoms of a sample should be included in the analysis of an x-ray pattern. By traditional diffractometry this is a very hard task to achieve especially in the case of polymers as such images could not be obtained because of weak coherent scattered beams scattering from the sample at a large (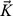), beyond 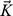
>12 Å^−1^.

However by applying amorphography 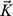 could be extended to large values and hence we obtained the precise reduced interference function, 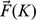, as previously shown for pure amorphous polyaniline [5], [12].

#### Fourier analyses of ρ(x,y,z)-Electron density expressed by the Fourier series

It is known that the intensity of diffracting materials, or the electron density ρ(x,y,z), varies with various substances. In the case of a crystalline substance the variation is periodic along any direction through the lattice. Therefore it is possible to describe electron distribution by a Fourier series with a structure factor 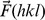 as a coefficient. The problem of determining the crystal structure or of determining the electron density, ρ(x,y,z), in the unit cell would be resolved if we could determine the structure factor, 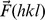, for all the nodes of the reciprocal lattice (Fourier space image). However in the x-ray diffraction pattern there is nothing apparent to indicate the value of 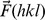. We therefore needed to know the phase associated with various structure factors, since any phase associated to 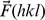 may lie between 0 and 2π for each spot of the x-ray diagram (image of Fourier space). Thus the magnitude of 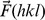 was obtained only from the integrated intensity I of a spot in an x-ray diagram, since 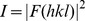 for each spot of the Fourier space image.

The question that arises is what information could we obtain from the magnitude of 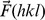. Patterson showed that it was possible to set up a Fourier series in which the coefficient 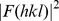
occurred and the information about the atomic position could be derived without making any assumption on the signs of this Fourier coefficients as required in a Fourier series of electron density ρ(x,y,z).

This mathematical function has a form similar to the for electron density, ρ(x,y,z) except that all phases, (φ_hkl_) set, are effectively zero. The Patterson function *P*(u,v,w) (or Patterson maps), like ρ(x,y,z), gives a three dimensional density distribution known as the Fourier transform of the diffracted intensity. The Patterson function is the average of the product of electron densities at two points (atoms) separated by a vector 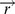
in the Patterson space. This function can be determined experimentally, (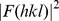 is simply a measure of intensity 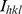 in scalar quantity) whereas the electron density cannot. In the Patterson space *P(u,v,w)* is zero, except when *u,v,w* are coordinates of a vector separating two atoms in an ideal crystal.

There is a striking analogy between the Patterson function, *P*(u,v,w), in crystals and in samples which are homogenous and disordered. In this latter case a Patterson function effectively is reduced to *the atomic distribution function,* 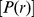, providing the probability of the centre of two atoms (m) and (n) lying on vector 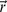; is the atomic distance. The numerical value of this function is chosen to be unity when all distances are *equally probable* [*P*(r) = 1]. This state is only possible for truly random assemblies of atoms, which means that any given atom does not have an influence on its neighbour and there is no interatomic interference between these atoms. Such an imaginary state was defined for a perfect gas, which could be considered as a theoretical standard for a state of disorder. If the gas is far from this idealised state even with a low density the interaction between the atoms can no longer be ignored, thus: *P*(r)≠1 results in fluctuations of the Patterson function.

This fluctuation expresses the existence of local order in atomic network of a homogenous substance, meaning that there are interferences of scattered waves of atoms. The scattering intensity from the sample was obtained by summing the amplitude (A) of scattering beam from N different atoms. 

Since: 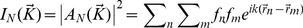
(1)


If each vector 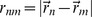 takes all possible orientations and we define 
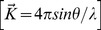
 as the scattering vector, we obtain a very general of the average intensity from an array of atoms, which take all orientations in K space.

Here, the intensity can be defined as the electron unit; I_eu_ = I/I_e_ where (I_e_) is the scattered intensity by free electrons (Thomson’s formula) [32].

Therefore we can calculate I_eu_ in the case of an isotropic distribution of atomic distances by: 
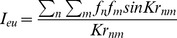
 (2) ***(The Debye scattering equation)***

As mentioned above, (f), is defined as an atomic scattering factor of each atom and |r_mn_| is the magnitude of distance between each atom from the other.

For a sample with one kind of atom 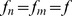 then 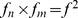 then:



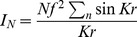
(3)Where N is the effective number of atoms in the sample

We define the interference function as 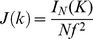
(4)


Hence the interference function that denotes the constructive interference can be written as



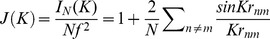
(5)


By inverting the experimental intensity function by means of the Fourier integral theorem we obtained the atomic distribution function *P*(r). This mathematical operation led to the discovery of a function, which shows ***the radial distribution of atoms*** surrounding any given atom in an isotropic liquid.

This is obtained from a Fourier analysis of the experimental x-ray scattering curve *(image of Fourier space)* and directly provides, the average number of atoms found at any distance from any given atom.

Therefore in this manner one is not making any “a priori” assumption for the structure of the substance.

When the radial distribution function such as 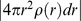
is introduced expressing the number of atoms between distance (r) and r+dr from any atom, the Debye scattering becomes;
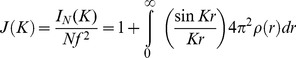
(6)Here *J(K)* is the interference function. If we define (*ρ_o_*) as a constant and the average density of atoms per unit volume (or macroscopic density) the above may be written as:



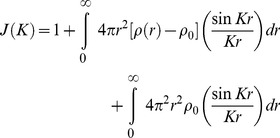
(7)The second integral represents the scattering of the liquid sample when it is truly homogenous. It can be neglected since it corresponds to the small angle scattering, which is really unresolvable from the direct beam if the sample is truly homogenous.

The first term of (7) can be written in another form:
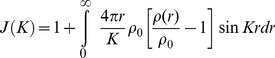
(8)As mentioned, above the Patterson function, *P*(r), in the case of a disordered material *will be reduced to the atomic distribution* or pair correlation function then:



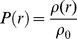
(9)Thus we obtain:
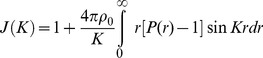
(10)Or
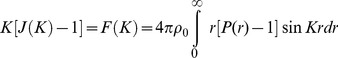
(11)Here, 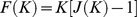 is defined as a reduced interference function.

Inverting this by the Fourier integral theorem, we obtain:
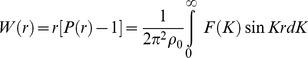
(12)

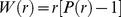
(13)


Then 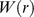 in (13) is defined as the *reduced radial distribution function*.

Zernicke and Prins [13] explained the occurrence of diffraction halos, which is a characteristic of scattering by liquids, and derived from this function. In this study we needed both the precise experimental data and the theoretical treatment proposed by Zernicke and Prins to represent the distribution of intensity of halos given by a liquid obtained from the real image of Fourier space of the sample.

By using (13) we obtained *W(r)* from the coherent diffracted intensity of sample *I_N_ (K)* through the reduced interference function *F(K)*. Also from the maxima (*r_max_*) of the *W(r)* curve we could obtain the average distance of n^th^ neighbours (d_n_). When the peaks of oscillation were well defined the ***average*** coordination number (Z_n_) to the n^th^ nearest neighbour was obtained by integration according to the follow expression:
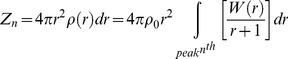
(14)


Equations (7) and (13) were applied to the monoatomic components since the scattering factor of carbon and oxygen was close and the x-ray scattering factor of hydrogen was negligible. In this case data was derived from one dimensional x-ray scattering. Hence, precise information about interatomic distances of the hydrogen atoms was not obtained. In practice, the interference function was derived from the experimental data *Ic (2θ)*. Measured intensities were modified according to the absorptions of x-rays by the sample in order to obtain the real intensity function, *Ic(2θ)*. This intensity was only characteristic of the coherent scattering process. The weak absorption correction was performed for angular variations using geometrical arguments.

It is known that in the reflection geometry the absorption corrected factor is independent of the diffraction angle; therefore in relative measurements corrections were unnecessary. Intensity measurements by transmission and reflection geometry were adjusted by overlapping the common angular domains. The difficulties in attaining a high quality experimental intensity, *Ic(K)*, were related directly to the K_max_ range. For a large K (K_max_, ≥20 Å^−1^) high precision was required to detect a weak diffraction beam by a polymer sample with a weak scattering power (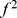). Due to this weak scattering power the elimination of an intense Compton radiation by classical diffractometry was a delicate procedure. These major difficulties, the elimination of Compton radiation experimentally and other classical obstacles were solved by anomalous diffractometry. By this method we obtained a near perfect reduced interference function from these data using the classical procedure of normalisation for disordered materials. This procedure of normalisation was originally introduced by Kaplow *et al*. [14] and later on developed in the Guinier School to study the structure of disordered materials [6], [15]. Normalisation consists of dividing the experimental intensity by the calculated gaseous scattering intensity and adjusting for the large value of (K≥20 Å^−1^). Small errors were detected and corrected by taking into an account the low r (Å) contribution to the reduced radial distribution function obtained by the Fourier transformation. By this method the experimental intensity was analytically corrected, with the assumption that the interatomic distances were not smaller than the nearest neighbouring distances

The first maximum peak of the *W(r)* curve indicated that the curve below the first peak of the reduced radial distribution function, *W(r)*, had a slope of (–1). Therefore, the accuracy of the experimental data was determined by investigating the behaviour of the curve below the first peak. If the curve in front of the first true peak had an appropriate characteristic, that is a slope close to −1, the data was considered to be acceptable.

By this method we succeeded in obtaining the total radial distribution function and partial atomic distribution function of polymers and previously various metallic alloys [6], [16], [17].

## Results and Discussion

The aim of this present study was to investigate whether the characterisation of such complex material with the polydisperse criterion of the molecular weight (M) (chain growth is controlled by probability) could be achieved. We therefore used anomalous diffractometry to determine the full real Fourier space image of PEG. Our results indicated two types of images; diffuse rings and lines superimposed on diffuse halos.

### Diffuse Rings

#### Real Image of Fourier Space by anomalous diffractometry for liquid PEG

These consisted of intense broad diffuse rings (principle peak) with some weak rings when molecular weight, (M) was 200, 300 and 600. Figure 6 shows that the general feature of the Fourier space image (reciprocal space) of these two liquids was the same at room temperature. At room temperature the principle peak was at its maximum at the scattering angle of 2θ = 7.6°. As mentioned previously the first attempt to analyse these x-ray diffuse scattering curves by Bragg’s law led to an incomplete conclusion resulting in the model of fragmentary crystals. However we obtained the real full image of Fourier space at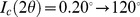 by anomalous diffractometry and this indicated the absence of diffuse scattering near the direct beam for liquid PEG (Figure 6B). The absence of diffuse scattering at a small angle of the x-ray curves from liquid PEG meant that large-scale inhomogeneity was not present in these three liquid samples at room temperature. Hence there was no definite preference for inter-particle distances (*d*) as computed by Ehrenfest’s (
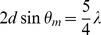
) such as the distribution shown as a major characteristic of x-ray diagrams of a very large diffuse halo at 2*θ* = 7.6°.

**Figure 5:**
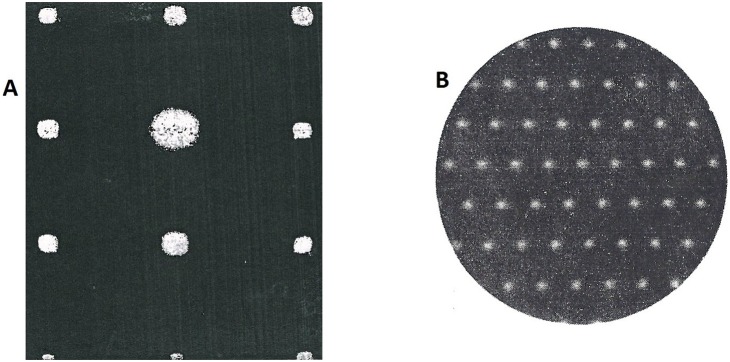
The Diffraction pattern. **A**-The fringes from two-dimensional optical gratings; the points show where the fringes of each grating intersect. **B**- Is the electron diffraction pattern of a single crystal of a thin gold film.

**Figure 6:**
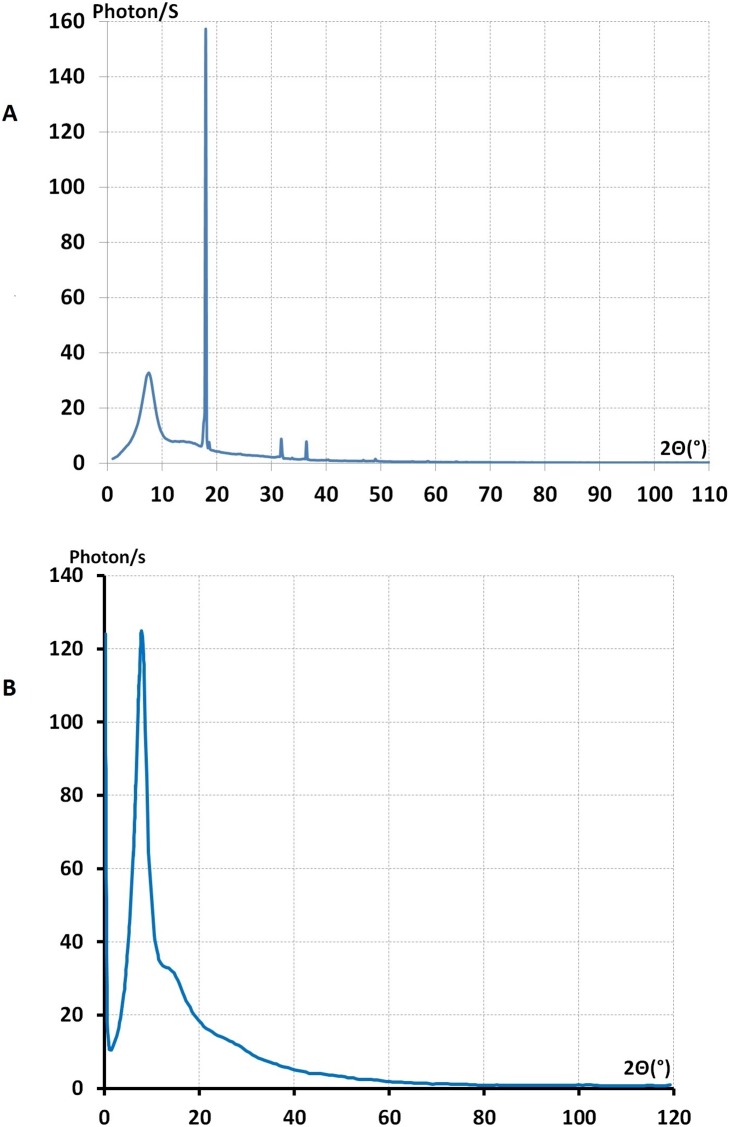
The full real image of Fourier space. **A**-The full real image of Fourier space of the ensemble of liquid (PEG)600, and sample holder, Ic(2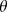). The sharp lines were the Bragg reflection from sample holder berylium for λ = 0.5609 Å. **B**-The full real image of Fourier space of liquid (PEG)200 at 0°C, Ic(2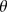).

We computed, using Ehrenfest’s equation, a mean distance between pairs of diffracting particles, as *d* = 5.25 Å for both (PEG)_300_ and (PEG)_600_ at room temperature. A value from Bragg’s (*d* = 4.22 Å) was obtained, meaning that a two-model system did not characterise these different liquids by their principle halo in the real image of Fourier space.

We postulated that the atomic arrangement of physical state of liquid and solid polymers was not related to their (n) value. That is the (n) value could not be considered as an invariant for the identification of liquid PEG polymers. To verify our hypothesis we obtained the full Fourier space image of both liquid and solid PEG with different (n) values.

The application of classical x-ray diffraction technique to determine structural information for polymers is limited. Therefore for analysis of liquid structures by the Fourier space image it is essential to investigate all positions and not just one or two peaks.

To study this anomalous diffractometry was used. As mentioned in the Methods this technique permits us to obtain a real full image of Fourier space with a very large K, associated to the analysis of radial-distribution method. The use of this method to determine the geometrical arrangements of atoms in atomic networks has been shown previously [4], [5], [10], [15], [17], [18].

Additionally, we cooled down *in situ* these three liquid PEG to study the structural evolution during temperature variations. We also studied PEG where M = 1000, to obtain a second type of Fourier space image of the solid-state polymer.

### The analysis and interpretation of interference functions *F(K*) of PEG liquids

**Figure 7:**
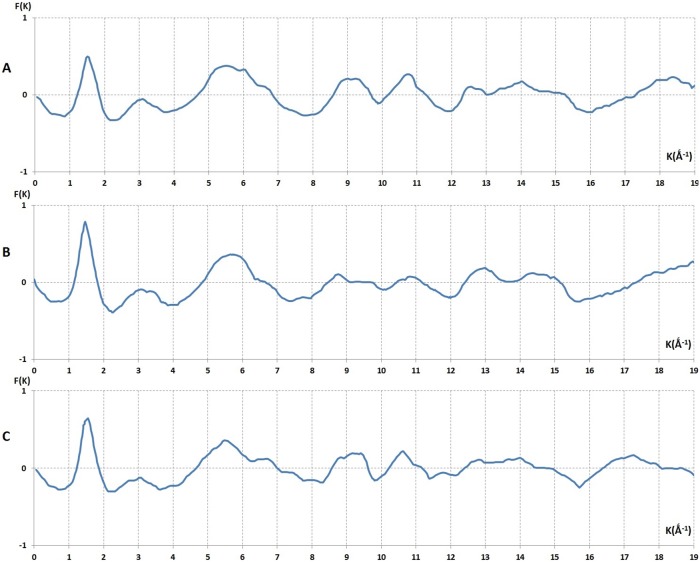
The reduced interference functions, *F(K)*, of liquid PEG derived from *I_c_*(2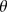). **A** and **B**, correspond to the samples (PEG)300 and (PEG)600 respectively at room temperature (19°C). **C** corresponds to (PEG)_200_ at 0°C. The feature of these curves were similar before 9 K Å^–1^ beyond the K = 9 Å^–1^. The curve obtained from (PEG)300 shows that the oscillation were more intense than *F(K)* of (PEG)600. In the case of liquid (PEG)200 at 0°C, the shoulder at 6.5 Å^–1^ were more evident than the other *F(K)* curves in **Figure C**.

Figure 7 displays, the reduced interference function, *F(K)*, of different PEG, liquids with different molecular weights, M = 200, 300, and 600. We defined, the *reduced* interference function as 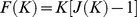 where 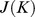
is the interference function as defined in the Methods.

For a perfect gas, an imaginary state of matter, a very small density was defined. The ratio of volume of atom to the average volume that it occupies is almost nil, hence 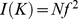. In this case scattering was similar to the scattering of an isolated atom. *F(K)* remains constant and equal to zero. Consequently the straight line in Figures 7A–C represents a reduced interference of a perfect gas. This line is considered as a form factor of this imaginary state and can be used as a reference for a perfect disordered state. Hence, the modulated feature of *F(K*) of PEG liquids indicated that the atoms in these liquids were tightly packed to form a local order known as a short range structure.

Previously x-ray diffraction studies of polymers were used to investigate the space packing of atoms to try to establish whether their geometry is regular or irregular.

Bernal [22] found that a periodic ordering in space was inconsistent with the properties of water and introduced for this the radial distribution method [*W(r)*] and other researchers, Debye, Warren and Eisentein have used this method to successfully determine the probability [19]–[21] function *W(r)* or the interatomic distance in the atomic network of various samples. However, this method could only be used if a high quality *F(K)* were achieved.

By using our method this proved to be possible and we showed that the principle peaks in the three *F(K)*s, of (PEG)_200_, (PEG)_300_ and (PEG)_600_ at room temperature were similar. The (K)_max_ position of each peak is K_max_ = 1.5 Å^−1^ and the half width maximum breath of three principle peaks in the three curves were also the same *ΔK* = 1.6 Å^−1^. These values may express the degree of atomic disorder or the randomness of the far atomic shells. In other words the profile of the principle peak was associated with the far atomic correlation in liquids. Signifying that liquid PEG at room temperature had a similar correlation for long-range order.

Hence we conclude that structural information that is based only on the molecular weight and the principle peak should not take centre stage for the characterisation of polymers as it has been suggested in many reports of polymer science. If we analyse the whole reduced interference function *F(K)* and not only the principle peak it can be seen that the principle peak of the interference curves of the three liquids are alike qualitatively. However, there are distinct quantitative differences between these three curves and these may help us to obtain structural information about the atomic assembly of the three liquids.

This led us to consider whether in liquid polymers there were any medium-range correlations, as in disordered solid substances. So we investigated the second and third peaks of different *F(K)* of PEG liquids at room temperature. The maximum position of the second peak was at the same position approximately at K_2_ = 3.4 Å^−1^ and their half width breath (FWHM) also was the same; ΔK_2_ = 3.1 Å^−1^. The third peak also had the same maximum position for the three curves at K_3_ = 5.7 Å^−1^. But at high values of K the behaviour of the third peak was different [Figure 7A, B]. For M = 300 a slight shoulder was observed. This is a most important effect, related to the variations of M appearing at high K values. This shoulder became even more evident at a high K value for M = 600 [Figure 7B].

The next step was to examine the way in which atomic arrangements change during transformation of the liquid into the solid state. When we cooled down (PEG)_200_ liquid to 0° it still remained in a liquid state. Under this condition the maximum of the principle peak did not change but was shifted from K′ = 1.50 Å^−1^ to the K_1_ = 1.57 Å^−1^. The most important modification in this curve was the splitting of the third shoulder into two separate peaks [K_4_ = 6.63 Å^−1^ and K′_4_ = 7.45 Å^–1^]. Whereas (PEG)_200_ remained in a liquid state, (PEG)_600_ and (PEG)_300_ transformed to the solid state at approximately 17°C and 10°C respectively. Figure 7A–C shows the effect of temperature variation on the third peak of the reduced interference function.

The general feature of *F(K)* curves were consistent with the theoretical interference functions derived from “Dense Random Packing” (DRP) models, which was originally put forward by Bernal using the Frank unit (icosahedral symmetry) as a fundamental irregularity in atomic network of liquids [3], [22], [23]. This resulted in constructing the DRP model for describing liquid structures.

The similarity of the experimental *F(K)* curves and the ratio of the third peak position to the second peak position plus the singularity of their shoulders suggests that the icosahedral packing model, i.e. the dense random packing model, is a reasonable approach for describing local order in the atomic network of liquid polymers. The reason for the above assumption is the presence of the shoulder on the second and third peak as well as the value obtained from the ratio of the third peak to the second peak position.

If we consider the *F(K)* derived from (PEG)_200_ at 0°C then the position of the shoulder was at K_4_ = 6.8 Å^−1^ and the ratio is: K_3_/K_2_ = 5.5/3.2 = 1.718. This value was recognised as a signature for the icosahedral DRP models. Beyond the shoulder there were two very large peaks at K = 9.5 Å^−1^ and K′ = 10.5 Å^−1^. The *F(K)* curves beyond these large peaks show a degree of disorder in this liquid which was similar to a perfect gas as the oscillations subside. The sensitivity of the shoulder to the temperature variation in the liquid polymer (PEG)_200_ was similar to that shown in previous studies of amorphous Pd-Si, and Fe-B. This was explained by interatomic fluctuations on the local topology meaning that a change was occurring in the degree of tetrahedral perfection in the DRP models. The geometrical model of Bernal was extended to describe the structure of amorphous metallic systems (A–B). The coordination of the atomic number of the nearest neighbours and interatomic distances, obtained experimentally when compared to those derived from the theoretical geometrical model, indicated the viability of the DRP model [24]–[26].

### Analysis of intensity in Fourier space by Fourier method: the analysis and interpretation of reduced radial distribution function, *W(r),* liquid PEG

By Fourier integral theorem we obtained precise information about the radial distribution of atoms surrounding any given atom in a liquid.

#### Structural determination by geometrical factor

**Figure 8:**
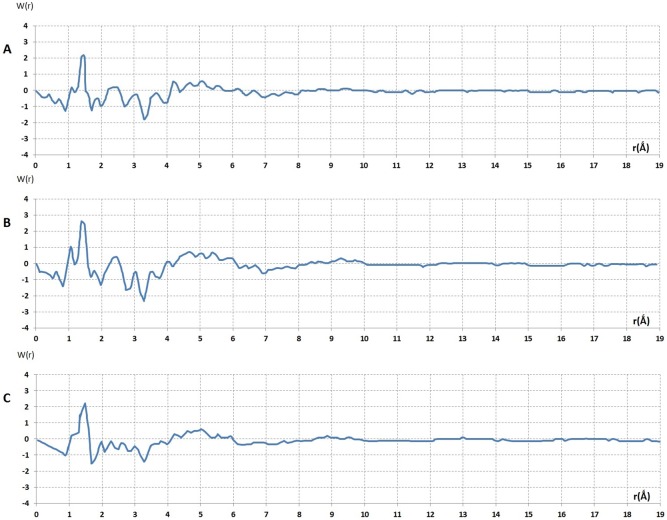
The reduced radial distribution functions of liquid, *W(r)*. These were derived from reduced interference function, *F(K)*, of (PEG)300 and (PEG)600 at room temperature and (PEG)200 at 0°C°Fig. (7.C).

Figure 8 shows the reduced radial distribution functions derived from the high quality reduced interference functions of liquid PEG. Generally the characteristics of these three curves were similar. The maxima of these curves corresponded to the distance between two atoms in the atomic network of these liquids. The first peak was sharp and well resolved. The sharpness of the peak indicates that the distance between two atoms was well defined and the area under the peak was directly attributed to the number of nearest atoms.

We neglected the scattering by hydrogen therefore we considered these liquids as a binary system (A–B) of carbon and oxygen atoms. We can write the empirical formula of liquid PEG unit as [C_x_-O_1_-_x_] where x = 62, 63 and 65 for M = 200, 300, 600 respectively.

Figure 8A shows *W(r)* derived from *F(K)* obtained from (PEG)_300_ at room temperature. The maximum at r_1_ = 1.48 Å can be attributed to the atomic diameter of C-C, where r_1_ = C_1_–C_2_ = 1.48 Å and its broadness Δr_1_ is 0.16 Å^.^ The second peak in the case of M = 300 and 600 was very broad and asymmetrical, Δr_2_ = 1.7 Å^.^ This means that the maxima of the *W(r)* curves overlapped. A broad peak area could not be used to determine the exact numbers of neighbouring atoms. At room temperature the estimated value related to the Δr_2_ indicated large fluctuations for inter-atomic distances in the liquid atomic network. This was not surprising since these broad peaks could be considered as a criterion of liquid polymers which have disordered structures. Figure 8C shows the radial distribution function, *W(r)*, derived from reduced interference function of (PEG)_200_ at 0°C (Figure 7C). Here the maximum of the second peak position had a splitting, contrary to the *W(r)* curves at room temperature, which were well resolved. The maximum at r_2_ = 2.57 Å with the splitting at r_3_ = 2.98 Å could be attributed to the atomic diameter r_1_ = C_1_–C_2_ and r_2_ = C_1_–C_3_ or the single bond length of second and third neighbours.

The singularity of dense random packing, in the hard sphere model, is the existence of diameters equivalent to 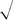
3σ, where (σ) is the atomic distance of C-C, the presence of ∼2 diameters and the absence of 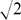 diameters. The distance of 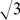
σ and ∼2 diameters corresponds to the two edges across sharing equilateral triangles and the second neighbour consists of three spheres separated by a common first neighbour along the line through the centre of three spheres. Such a configuration of three spheres in a straight line, was observed by Bernal in his physical model for the structure of liquids [3]. The positions of the maxima in the *W(r)* curve, with the ratios of 
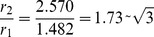
 and 
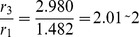
, corresponded to a tetrahedron geometry. This is the most stable configuration of a cluster of four atoms, which provides a pseudo-nucleus or a seed in the liquid state.

This type of atomic arrangement must be different from cubic or hexagonal dense packing, which is defined by the 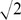 diameter [the body diagonal of a regular octahedron]. The absence of this maximum at 1.48
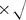
2 = 2.09 Å in a well-resolved region excluded the periodic structure of f.c.c. and h.c.p., because at this position there was a minimum instead of maximum in *W(r)* curve (Figure 8A–C).

It is known that 20 regular tetrahedra pack, naturally, with a slight distortion, to form another geometrical configuration with icosahedral symmetry (Frank Unit). This regular arrangement possesses six [five fold] symmetry axes, which do not allow the formation of a crystalline lattice for filling up the space.

With such experimental results obtained from *W(r)* the DRP model would be a reasonable approach to describe the structure of (PEG)_200_ liquid. Thus, it was possible to estimate the positions of the non-resolved maxima in *W(r)* derived from liquid PEG at room temperature. Regarding the estimated positions of the maxima obtained by analogy the corrected positions of maxima could be defined by ratios of r_2_/r_1_ and r_3_/r_1_ from the (PEG)_300_ and (PEG)_600_ *W(r)* curves. This would determine whether the positions of the maxima corresponded to the signature of dense random packing models.

### The topology in dense packing of the Frank Unit

#### The geometrical model

In the mathematical model of crystals the atomic arrangement are determined unequivocally by the topology of space lattice. In the case of icosahedra (Frank unit) assembly, detailed connections between atoms are not, uniquely defined. In icosahedra assembly, the topology of DRPs is not uniquely fixed and there is space for central atoms to be configured in an arbitrary way. Each approach provides a different mathematical model with different theoretical *W(r)* curves. Other authors have suggested that to obtain an adequate theoretical model with an adequate packing density, the fit with the short-range order of experimental radial distribution function curve *W(r)*.

We observed that on the left hand side of the principle peak (1.48 Å) there were maxima at 1.189 Å for (PEG)_200_ at 0°C, 1.079 Å and 1.094 Å for PEG_300_ and PEG_600_ respectively, which was surprisingly much smaller than the covalent length of a single carbon-carbon bond (1.48 Å).We also observed other peaks on the right hand side of the principle peak in each *W(r)* curve of these three liquid samples (Figure 8A–C). These peaks correspond to the distances (r_2_′ is equal to C_1_ = C_2_ where r_2_′ = 1.990 Å, 1.788 Å and 1.788 Å) in the case of (PEG)_200_, (PEG)_300_ and (PEG)_600_ respectively. The ratios of these two interatomic distances were about 1.63 (Table 1) and the positions of split peak (r_3_′′) to the first peak (r_1_′′) was about (r_3_′′/r_1_′′)∼2 (Table 1). This was similar to the value for (PEG)_200_ at 0°C (Tables 1 and 2). These results suggest that these atoms formed another tetrahedral arrangement. Moreover, there were other maxima at r_1_′′ = 1.378 Å that were not well resolved from the principle peak (1.48 Å) in *W(r)* curves derived from *F(K)* for (PEG)_200_, (PEG)_300_ and (PEG)_600_. This distance could be attributed to the interatomic distance of C-O atoms. The ratio of r_2_′′/r_1_′′ = 2.380/1.378 = 1.720 and r_3_′′/r_1_′′ = 1.97 in these samples (Table 1).

**Table 1:** The interatomic distance between the different atoms of (PEG)200, (PEG)_300_ and (PEG)_600_ at liquid state.

r_m_	r_1_(Å)	r_2_(Å)	r_3_(Å)	r_2_/r_1 _g.f	r_3_/r_1 _g.f	r′_1_(Å)	r′_2_(Å)	r′_3_(Å)	r′_2_/r′_1 _g.f	r′_3_/r′_1 _g.f	r′′_1_(Å)	r′′_2_(Å)	r′′_3_(Å)	r′′_2_/r′′_1 _g.f	r′′_3_/r′′_1 _g.f	t°C
(PEG)_200_	1.482	2.570	2.99	1.73	2.01	1.189	1.99	2.286	1.67	1.92	1.378	2.390	2.71	1.73	1.97	0°
(PEG)_300_	1.49	2.469	3.09	1.66	2.03	1.079	1.788	2.98	1.66	2.00	1.378	2.348	2.79	1.70	2.02	19°
(PEG)_600_	1.473	2.473	2.98	1.68	2.02	1.095	1.788	2,28	1.63	2.08	1.378	2.348	2.87	1.72	2.08	19°

r_m_: The position of the peaks maxima in the total radial distribution function, *W(r)*, r_m_/r_1_,: geometrical factor (g.f).

The variation of g.f conserves the singularity of D.R.P. In this model; three atoms *(* ***trio***) are in hard contact, the forth one (***the apex***) is free to find its exact position. In these liquids the different values of (g.f) of single bond; C-C, C-O and double bond C = C in a pentagonal chain is shown.

The experiment indicates the fluctuation of the values of the geometrical factors between √3 = (1.7320) (singularity of regular tetrahedron) and √8**/**√3 = 1.6329 diameter (bi-pyramid configuration). The r_3_/r_1_ shows the effect of medium range order on the local topology of the atomic network for each sample.

**Table 2:** The interatomic distances between the different atoms of (PEG)_200_ liquid state in different temperature.

	Å	Å	Å	g.f		Å	Å	Å	g.f		Å	Å	Å	g.f		Temperature
r_m_	r_1_	r_2_	r_3_	r_2_/r_1_	r_3_/r_1_	r′_1_	r′_2_	r′_3_	r′_2_/r′_1_	r′_3_/r′_1_	r′′_1_	r′′_2_	r′′_3_	r′′_2_/r′′_1_	r′′_3_/r′′_1_	
(PEG)200	1.593	2.750	3.18	1.75	2.00	1.189	2.00	2.300	1.68	1.93	1.489	2.51	3.0	1.69	2.00	**10°C liquid**
(PEG)200	1.583	2.788	3.09	1.76	1.95	1.189	1.90	2.378	1.76	2.0	1.280	2.28	2.51	1.78	1.96	**2.5°C liquid**
(PEG)200	1.483	2.570	2.99	1.73	2.02	1.189	1.99	2.286	1.67	1.92	1.378	2.39	2.71	1.73	1.97	**0°C liquid**

r_m_: The maximum position of *W(r)* curves derived from interference function *F(K)*, obtained from (PEG)_200_ at different temperatures (*in- situ)*. The local topology in the atomic arrangement of each sample is determined when the temperature has changed.

r_m_/r_1_: geometrical factor (g.f).

In summary, from analysing the *W(r)* curves derived from reduced interference functions of each liquid at room temperature the geometrical factor was identified for each tetrahedron. This defined the geometry of the short-range order. This heap of molecules characterised by the geometrical factor could be considered as a pseudo-nucleus “seed” in close packed liquid models.

These results led to investigating the absence of the maximum corresponding to the first neighbouring interatomic distance of [O-O].

**Figure 9:**
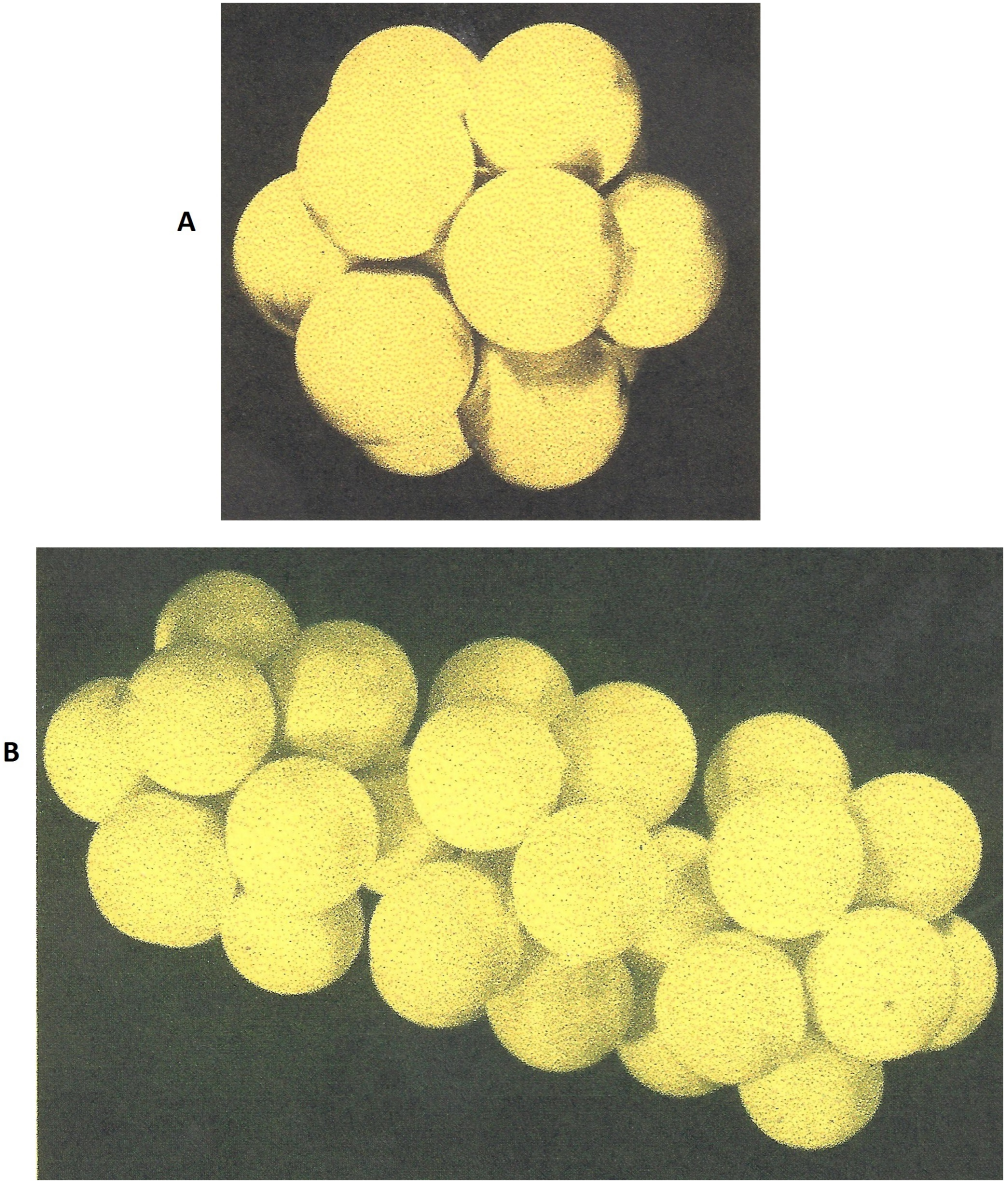
The Frank unit. **A** The irregular unit cell is based on the icosahedral packing of 12 atoms surround a central atom. The latter atoms are not in contact with other atoms. **B -** The pentagonal chain. This chain forms by joining the Frank unit with an icosohadral symmetry with homo-atom restriction of the nearest neighbouring atoms.

This was explained by considering the samples as super-cooled liquids, the Frank unit with icosahedra symmetry links. Hume-Rothery and Anderson [27], suggested a pentagonal chain model to describe the liquid structure. They considered the Frank unit (Figure 9A) with an icosahedral symmetry in which the central sphere is in contact with 12 neighbours but the latter is not quite in contact with one another. This Frank unit may join together in chains, such that one sphere is held in common between two adjacent irregular units to form a liquid chain with the configuration of …1-5-1-5-1… where each central sphere is in contact with two sets of five spheres and with one sphere above and one below but the side spheres are not in contact with each other (icosahedra symmetry) (Figure 9 B).

This chain was named by Hume-Rothery as a pentagonal chain. In the case of binary systems, when solute [B] is added to a liquid A [carbon] atoms at the centre of Frank unit, B [O] has the tendency to prefer [A] rather than [B] neighbours. However, we cannot substitute more than one B atom in this Frank unit introducing B-B [O-O] as direct neighbours. Thus, instead of pentagonal chains in “super-cooled” liquids, we have cubic or hexagonal close packing. This assembly would provide nuclei for crystallisation growth by avoiding the Frank units formation. Such liquids where it is possible to avoid B-B contacts are built by stacking sequence of layers of 1-5-**1**-5-1-5- **1**-5-1-5-**1**…. where **1** refers to the solute atoms B [O] [27].

#### Chemical Ordering (electronic structure)

Table 1 shows that the geometrical factors are more fundamental criteria than the value of the atomic diameter or Goldschmidt atomic radius in the structural study of polymers [6], [28]. These geometrical factors determine the local geometry of irregularity in the atomic structure of liquid polymers. Moreover, the interpretation of the Fourier space image of these liquids, for the first time, indicated how the Frank unit with an icosohedral symmetry is linked together to form a pentagonal chain with a chemical ordering.

The chemical ordering in these liquids was justified by first the absence of the O-O at a first neighbouring distance, and secondly the existence of other maxima in the *W(r)* curves, from these liquids. These maxima may be attributed to the C-C bond length (r_m_), an interatomic distance shorter than single bond length of C-C at 1.48 Å. As in the case of polyethylene, PEG also had an interatomic distance shorter than single bond length of C-C (1.48 Å) [6]. This was attributed to the double bond covalent radius, derived from quantum mechanics considerations. Resonance energy effects explain the shortness of this interatomic distance.

Pauling proposed [29] the following empirical to express the relation of atomic radius to the bond number (n). Pauling’s is:
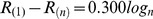
(15)Here, 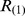 is the covalent radius of a normal single bond, (atomic radius) and 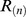
is the apparent radius (Pauling single bond radius) where “single bond “(ν) resonates among N positions of closest neighbours. The bond number is defined: n = ν/N. Here each atom contributes one electron and they are shared equally between the two atoms. This expresses the change in a single covalent bond radius of an atom with the change in bond number (n). With this fundamental idea Pauling pointed out the origin and the condition of the extra stabilisation is due to the resonance energy of a strong bond with a smaller interatomic distance [1.079 Å, 1.095 Å].

The evidence of the shorter bond-length r′_1_ = 1.070 Å and 1.095 Å (Table 1) may be equivalent to the radius of double bond carbon atoms in the network of (PEG)_ 300_ and (PEG)_ 600_ liquid respectively. The results of structural studies of different liquids, PEG with different molecular weight at room temperature, illustrated the predominance of short-range order identification, which in turn indicated the geometry of local order and chemical ordering. The *W(r)* of (PEG)_200_ near the freezing region (about 0°C) indicated that the bond-length fluctuated to affect the geometrical factors at different temperatures (Table 2), consequently to modify the degree of regularity of a tetrahedron or the degree of icosohedrality of molecular heaps in a liquid polymer. This may explain how liquid polymers transform into solid-states. Hence, at freezing temperatures they cannot be analysed as crystals.

By analysing radial distribution function, *W(r)*, it was shown that this phenomenon, by missing a maximum corresponds to 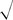
2 diameters in *W(r)* of (PEG)_200_ at 10, 2.8 and 0°C. However, freezing such a liquid would be transformed to the solid state. This transformation could be interpreted by a change in the morphology of pentagonal chain. The pentagonal chains prevented the formation of crystalline nucleus and induced a solid with an intermediary order state.

We used anomalous diffractometry to obtain the full image of Fourier space of these solids. The full image consisted of lines super-imposed on the coherent diffuse scattering, similar to that found in a previous study of metallic alloys such as Al_6_Mn (quasi-crystalline material [4], [30], [31].

Our next strategy was to therefore solidify liquid PEG in different ways and investigate their atomic structures, which would enable us to understand how these super-cooled liquids became solids.

Table 2 indicates the effect of isothermal cooling, on the molecular heaps in PEG liquids, before freezing. It seems that the atoms were free to move to change their inter-atomic distances however they maintained the singularity of the dense random packing of the Frank unit.

### The interpretation of Fourier space image of solid PEG

We have shed light on the atomic structure of liquid PEG which belongs to the series of polyethers, [(CH_2_)m-O]n when m = 2. We determined the atomic structure of solid polyethylene [m = ∞] as being an intermediate state of order [6]. We showed that the Fourier space image of these substances consisted of selective lines superimposed on the coherent background. For analysing the image a crucial step was the separation of the lines from the coherent background.

We applied our separation procedure, by which the Fourier space image was numerically resolved into coherent halos and lines, since it was possible to obtain the full real image of reciprocal space by anomalous diffractometry. The interpretation of this image was the intersection of reciprocal space with a sphere of reflections (Ewald’s construction) [32]. Figure 4 shows the possibility of separation of the coherent diffuse scattering halos from the selective lines.

### Analysis: lines superimposed on diffuse halos

By cooling down these liquid samples, we obtained a second type of Fourier space image, which consisted of lines superimposed on coherent diffuse halos. Although the Fourier space image of these three liquids at room temperature was similar, their temperature of solidification was different.

We obtained the second type of image (b) in the case of (PEG)600 at about 14–17°C and for (PEG)300 at about 10°C. The sample with molecular weight M = 200 at 0°C still remained in a liquid state. But the full image of Fourier space obtained by anomalous diffractometry indicated a structural modification with temperature change. This evolution may appear in form of a pre-peak, which can be considered as a signature for structural evolution.

**Figure 10:**
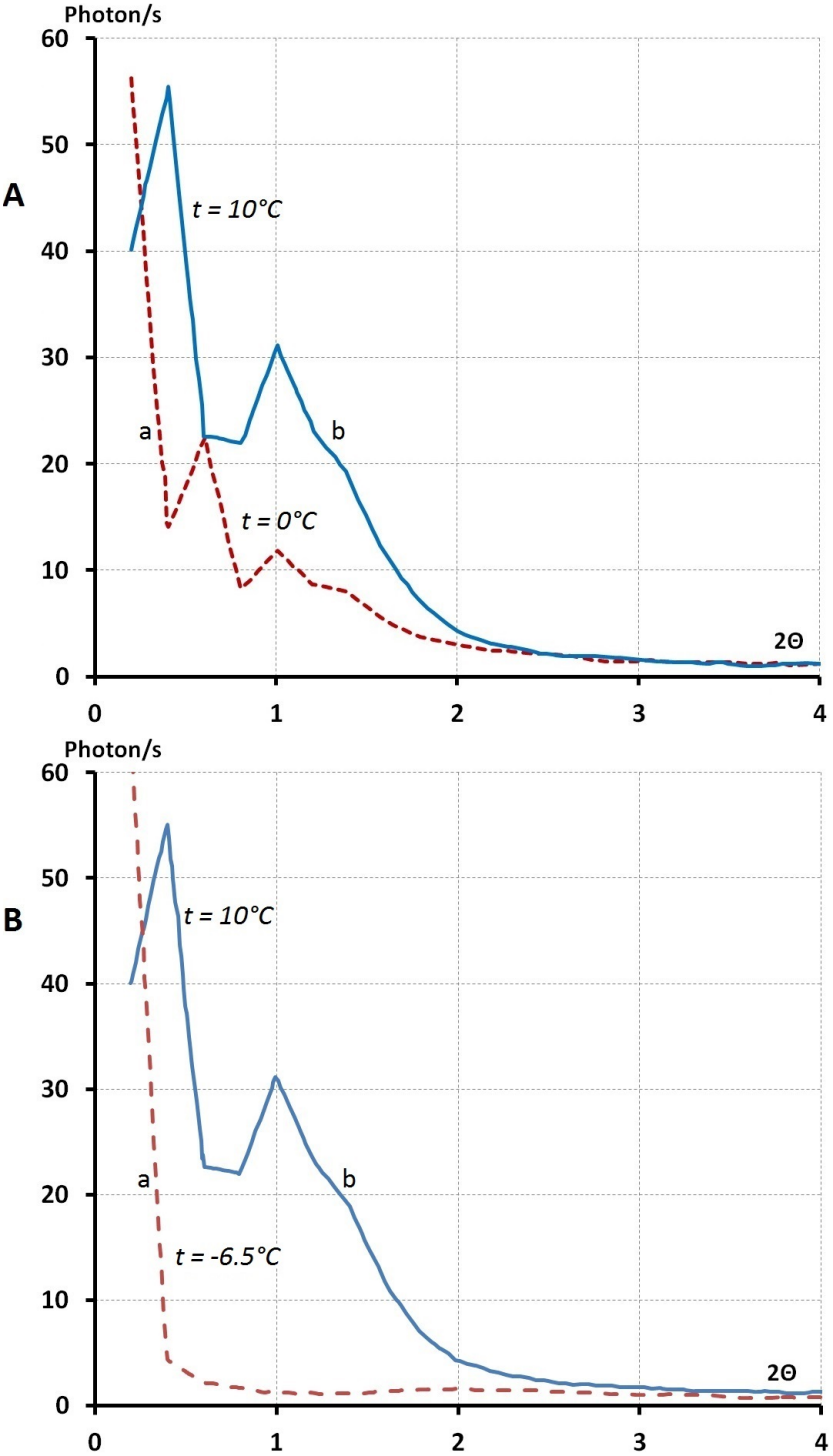
The small angle lines observed at full real image of Fourier space (2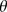 = 0.2–120°C; λ = 0.5609 Å^−1^) of liquid (PEG)200 at different temperature. **A-** liquid (PEG)200 at 0°C wavelength λ = 1.62 A°; b- Liquid (PEG)200 at 10°C λ = 1.62 A° This figures shows, the position of lines (2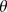) at a small angle where it was changed by varying the temperature from t = 0 to 10 degree C° for liquid (PEG)200. **B-** The small angle diagrams for liquid and solid (PEG)200 at 10° and −6 C° λ = 1.62 Å. **a-**The small angle lines from solid (PEG)200 at −6°C λ = 1.62 A° b- The small angle lines from liquid (PEG)200 at 10° and 0°C.

Figure 10. A indicates these pre-peaks [at **10°C**, 2θ = 0.4°and 1°; between **4.8°C–2.8°C**, 2θ = 0.6, 1.2, 1.4,1.8, and at **0°C**, 2θ = 0.4°, 1°]. For λ = 1.62 Å.

When the (PEG)_200_ liquid transformed to a solid the pre-peaks disappeared (Figure 10B) and we obtained the second type (a) of Fourier space image as in the case of (PEG)_300_ and (PEG)_600_. This diagram (b) generally has been analysed by over-simplification of the two phase model. In this case the coherent background was excluded and scarce lines were indexed as a Bragg reflections for polymer structural identification.

We next investigated PEG with a molecular weight of M = 1000, which was also a solid polymer. Its Fourier space images were similar to the image of solid samples obtained by cooling *in situ* or quenching. In polymer science, it was suggested that these lines were reflected by the crystalline part and the coherent diffuse scattering by the amorphous part.

**Figure 11:**
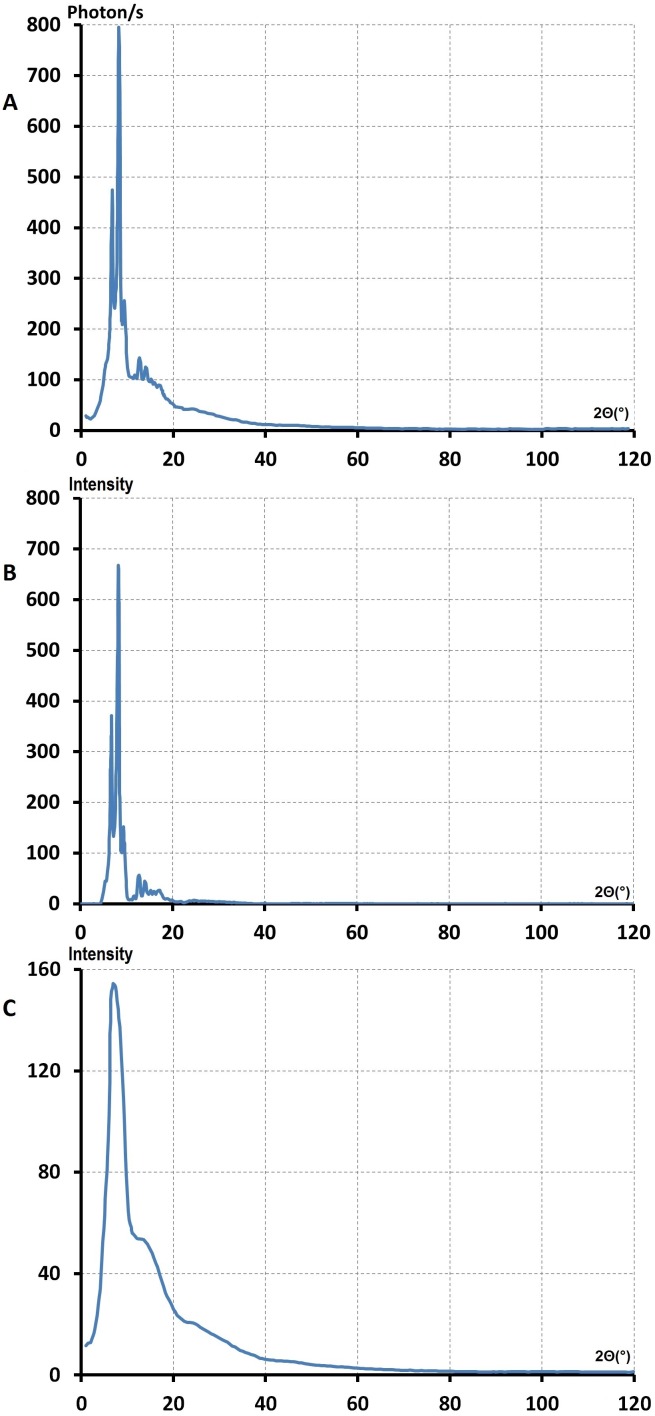
The full real image of Fourier space of solid (PEG)1000 at room temperature. (Solidification without heat exchange). **A-** This figure shows that selective lines were superimposed on the coherent diffuse scattering as the one solidified with heat exchanging **B-** Is(2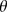) the lines spectra separated from coherent diffuse scattering. **C-** Ic(2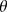) the coherent x-ray diffuse scattering after separation from selective lines λ = 0.5609 Å^1^.

However, for M = 1000, the x-ray diagrams behaved the same as other solid samples with M = 200, 300 or 600 but *without heat exchange* (Figure 11A). Therefore the outcome of these comparisons was that the two-phase system or crystal defect models could not be used as a “theoretical model” for interpreting the atomic arrangement of solid PEG.

The comparison of these experimental results with the theory of solidification as an atomic process poses the question: how are these apparent diversities of the physical structure related to the atomic arrangement of PEG?

Even by using high-resolution anomalous diffractometry, we obtained similar x-ray patterns for PEG, with molecular weights ranging from 200, 300, and 600. We may conclude that molecular weight may not be the only essential criterion for structural determination and it is related to a qualitative observation, especially when only the principle peak from the full real image of Fourier space is taken into consideration.

The x-ray pattern proved to be very similar to the microcrystalline aggregates e.g. Ni (Figure 2) so it was analysed accordingly. The position of each selective line was identified by deducing the inter planer spacing (d) corresponding to the family of (hkl) planes. Table 3 indicates a set of [d] spacings calculated from Bragg’s for all the lines. Care was taken for measuring the weak lines at small angle (θ) a necessary pre-request for structural determination of unknown structures. This was identified by the trial method, looking for a unit cell between a seven-crystal system (a Bravais lattice). The ease by which this direct method can be applied depended on the number of variables. For instance a cubic system with one variable (a) has one lattice parameter.

**Table 3:** Line spectra of (PEG)_200_ in Solid State.

Nr.	2θ degree	d Å	Intensity
**1**	1.40	22.480	0.60
**2**	7.00	4.590	246
**3**	8.00	4.020	133
**4**	8.81	3.650	101.5
**5**	9.80	3.280	3.70
**6**	10.59	3.039	3.36
**7**	12.00	2.680	3.17
**8**	12.39	2.600	4.40
**9**	12.78	2.590	5.20
**10**	13.61	2.35 (Al)	53.08
**11**	14.00	2.300	6.06
**12**	15.21	2.120	12.30
**13**	15.77	2.049 (Al)	28.60
**14**	16.19	1.990	40.70
**15**	17.38	1.930	6.130
**16**	18.57	1.730	16.80
**17**	19.17	1.690	3.00
**18**	20.00	1.610	1.20
**19**	20.40	1.599	0.50
**20**	20.80	1.553	2.18
**21**	21.59	1.490	2.60
**22**	22.40	1.440 (Al)	6.60
**23**	23.40	1.340	10.20
**24**	24.00	1.340	10.80
**25**	25.00	1.290	10.75
**26**	26.40	1.267	7.00
**27**	27.60	1.258	4.30
**28**	28.40	1.148	5.40
**29**	30.00	1.083	0.85
**30**	30.40	1.069	0.99

The positions of the lines spectrum, *2θ,* separated from coherent diffuse scattering of (PEG)_200_ cooling to the −8°c, *in-situ*,. The values of (d) have been, calculated by reduced of diffraction *grating*: 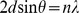
_._

Here λ = 0.5609 Å (λkα Ag), *θ* and I respectively are the position of selective lines, I is the relative intensity of lines after separation the lines from the coherent diffuse scattering.

The d_(hkl)_ = 2.35_(111)_, (2.49)_(200)_, (1.44)_(220)_, (1.38)_(331)_Å are considered as the Bragg reflections from sample holder.

To identify the crystal structure the agreement between the calculated and the experimental intensities was essential. For such a comparison we needed to know the atomic position within the proposed unit cell (mathematical model). The structure of solid PEG was too complex to be solved only by the traditional method. Despite this complexity the unit cell of PEG solid was first identified as monoclinic, with lattice parameters a = 8.16 Å b = 12.99 Å c = 19 Å and β = 126.5°. The same group proposed a triclinic unit cell with following parameters a = 4.71 Å b = 4.44 Å c = 7.12 Å and α = 62.8°, β = 93.2° and γ = 114.4° [33]. The authors mentioned clearly, the missing lines in experimental spectrum and had doubts on the correct values of intensity of each line. This discrepancy was caused by the oversimplification of the structural characterisation. Furthermore, our previous work on metallic alloys [30] showed that the degree of crystallinity could not be the main parameter for characterising these complex materials. Consequently for determining the atomic arrangement, the information “out-side” of the reciprocal lattice point should be considered in order to understand the correlation of properties with the structure of substances. Only by analysing the full real image of reciprocal space the nature of the irregular clusters in the atomic network, of these substance could be identified. That means, when freezing a liquid, the liquid does not always or naturally became solid with crystalline grains.

We have shown that the presence of selective lines, observed from the Fourier space image, did not illustrate the presence of crystalline grains. Figure 5A is from two-dimensional optical gratings with exactly the same feature of full image of Fourier space of crystalline materials (Figure 5B). Indexing such lines or sharp points is not a reasonable solution for determining the structural details and correlating them with the physical proprieties of substances.

**Figure 12:**
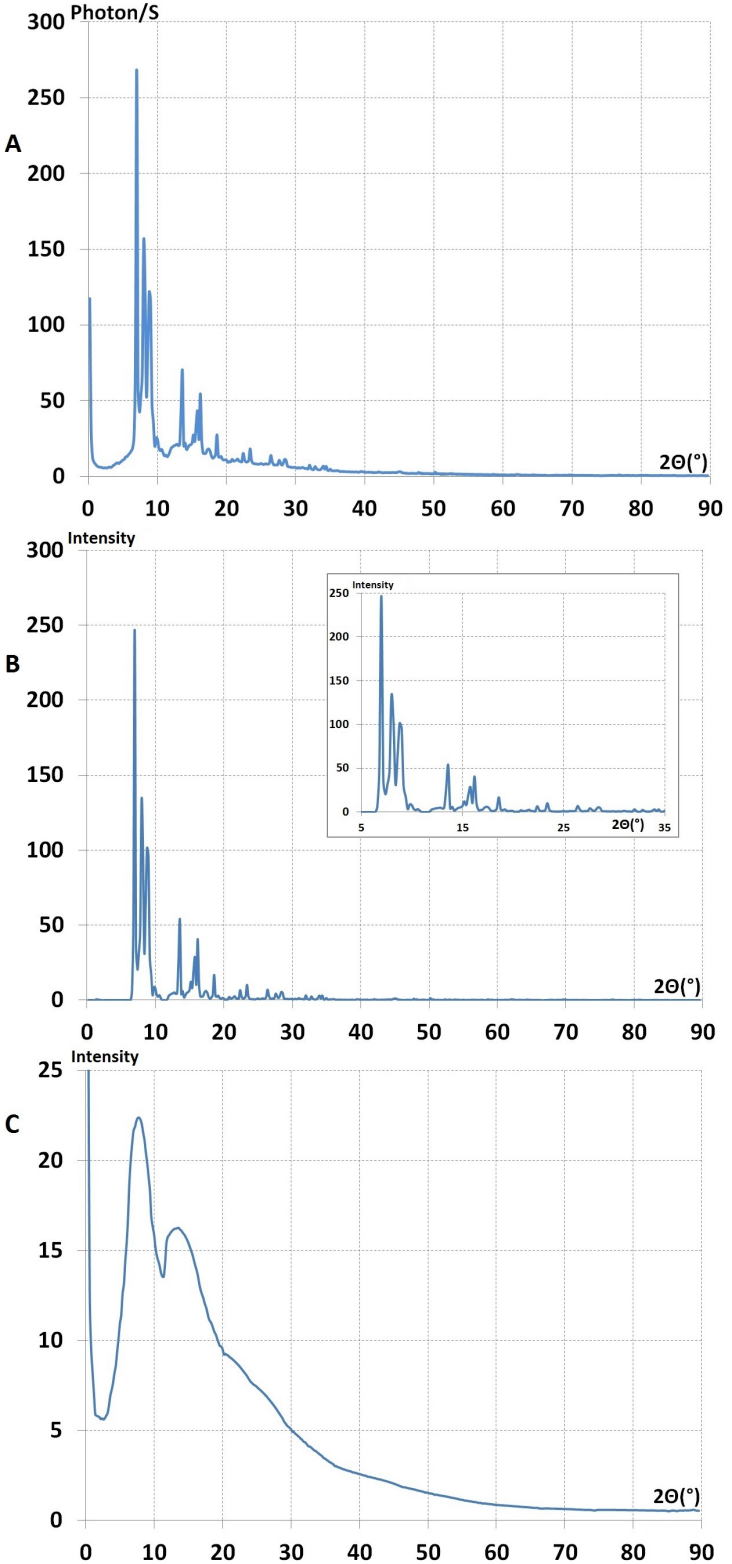
The full real image of Fourier space of solid (PEG)200. This image indicates selective lines were superimposed on the coherent diffuse scattering**. A**-This sample was obtained from, cooling down liquid (PEG) 200 *in-situ* to −6°C. Solidification with heat exchanging occurred.We show that when (PEG)200 was transformed to the solid state, the small angle lines disappeared (Fig. 10B). **B and C** show lines spectra and coherent diffuse scatterings after the separation of lines from the coherent diffuse scattering λ = 0.5609 Å^−1^.

It is possible to solidify PEG liquids without exchanging heat or by exchanging heat that is cooling (PEG)_200_ *in-situ* or quenching it in liquid nitrogen. In this case the liquid transferred to a solid state. In the case of solids the full real image of Fourier space consisted of lines superimposed on the coherent diffuse scattering (Figure 12A).

In classical physics the solidification of liquid substances that are small molecules such as metals and alloys occurs via nucleation and growth accompanied with a definite quantity of energy: the latent heat at the fixed freezing temperature Tc (Clapeyron Equation).

However, when (PEG)_200_ was cooled from room temperature to −20°C the solidification did not follow the classical process. The full real image of the Fourier space at 10°C, 2.5°C and 0°C before and after solidification was investigated. The analysis of these images using the radial distribution method showed that the liquid PEG did not transform to a crystalline phase at any time during the cooling process. On the contrary the image of the Fourier space of the solid state PEG consisted always of lines superimposed on a coherent background.

Taking all these together we have shown that the analysis of lines spectra alone was not sufficient for obtaining structural information. Therefore it was essential to analyse the second part of reciprocal space (coherent diffuse scattering) for determining the precise atomic arrangements of solid PEG during solidification without and with heat exchange.

### Analysis and interpretation of coherent continuous diffuse spectra

Figures 12C and 11C show the intensity of coherent diffuse scattering. Ic (2θ) is scattered by solid PEG after separating the lines from the coherent diffuse background. These curves indicate the form factors of the atoms in reciprocal space. The first halo in the Ic (2θ) shows the presence of a short-range correlation as described in the case of liquid PEG.

#### Interference functions derived from form factor of solid PEG

**Figure 13:**
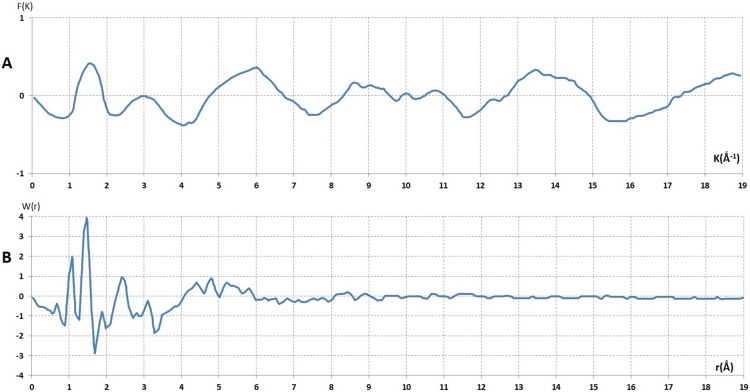
Analysis of coherent diffuse scattering from (PEG)1000. **A**- The reduce interference function *F(K)* derived from coherent diffuse scattering *I_c_*(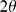) Fig. 12C. **B**- The reduce radial distribution from interference function of (PEG)1000, *F(K)* Fig. 13A.

***Solid state PEG without heat exchange: (PEG)1000:*** The comparison of reduced interference function, *F(K)*, derived from intensity of coherent diffuse scattering of (PEG)1000 (Figure 13A) separated from the lines by our analytical procedure with *F(K)* curves of PEG liquids indicated an essential agreement in behaviour despite the differences in profile and the full-width at half maximum [FWHM] of their principle peak. By measuring [FWHM] of the principle peak of solid (PEG)1000 we obtained ΔK = 2 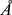 ^−1^ whereas in liquids the value was Δ Kliquid = 1.5 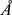 ^−1^.This increased value of ΔK = 2 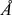 ^−1^ to the ΔKliquid = 1.5 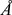 ^−1^ revealed the degree of atomic disorder: the solid state atomic disorder was greater than the liquid state of PEG. When comparing the behaviour of the second peak and the third peak of *F(K)* with the liquid’s *F(K)* we observed that the peaks were related to the medium range order.

This comparison indicates clearly that the dense random packing of hard sphere model could be adopted for the atomic arrangement of solid (PEG)1000, similar to liquid PEG. The ratio of third peak position, K3, to the second peak position, K2, was about ∼1.72 (Figure 13A).

This value is known as a signature of packing a hard sphere with an icosohedral symmetry. Here the Frank unit cluster has a fundamental irregularity in the network of solid (PEG)1000. Hence the comparison of the experimental *F(K)* of liquids and solids indicated a strongly that the conversion of liquid to solid state did not involve exchanging heat therefore could not be considered as a phase transition. Thus the solidification process involved only a substantial rearrangement of the Frank unit with an aberration in its crystal symmetry. This rearrangement could be attained only by deriving the radial distribution function *W(r)* from Fourier transformation of *F(K)*.

Figure 13B shows the radial distribution function of (PEG)1000. From the maxima of this curve the bond length of different atoms the single bond of C-C, O-O and C-O was deduced. The maximum of r_1_ = C_1_-C_2_ = 1.473 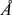 was attributed to the single bond of C-C. This value is different from the bond length of C-C in the crystal chain-model of diamond which is C1-C2 = 1.5433 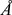 [6]. There were two other maxima at the left hand side of this principle peak. The one which was not well resolved from the principle peak was attributed to C-O r_1_ = C1-O = 1.378 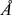. The second peak was attributed to C-C and the splits in two sub-peaks were r_2_ = 2.391 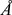 and r_3_ = 2.880 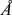.

The relative position of r2/r1 = 2.391/1.473 = 1.626∼1.63 = 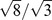 and r_3_/r_1_ = 1.955∼2.

These two values with the splitting of the second peak are the criterion of dense random packing models. This is the characteristic of structural features of non-crystalline materials. The same arrangements was adopted for C-O chains r3′′/r1′′ = 1.65 and r3′′/r1′′ = 1.995 [Table 4]. By studying carefully the radial distribution function, *W(r)*, we observed that maxima could not be attributed to the distances of O-O of each first neighbouring atom. The missing O-O in the first neighbouring atom suggested that the Frank unit with icosohedral symmetry was linked together to form pentagonal chains. The maxima r′_1_ = 1.094 Å and r′_2_ = 1.883 Å of *W(r)* corresponded to the double bond distances of C-C in liquid PEG at room temperature. The ratios of r′_2_/r′_1_ = 1.72 and r′_3_/r′_1_ = 1.99 indicated the tetrahedral geometry for double bond C = C. In the case of formation of solid (PEG)_ 1000_ without heat exchange, the *W(r)* indicated the absence of a maximum peak at 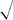
2
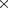
1.473 = 2.083 Å. The lack of this maximum at 2.08 diameter indicated an absence of fcc or hcp clusters in the atomic network of (PEG)_1000_.

**Table 4:** The interatomic distance between the different atoms of PEG solid state M = 200 and 1000.

r_m_ Å	r_1_	r_2_	r_3_	r_2_/r_1_	r_3_/r_1_	r′_1_	r′_2_	r′_3_	r′_2_/r′_1_	r′_3_/r_1_	r’‘_1_	r’‘_2_	r’‘_3_	r’‘_2_/r_1_	r’‘_3_/r_1_	Temperature
(PEG)_200_	1.558	2.690	3.00	1.73	1.92	1.189	2.000	2.24	1.68	1.92	1.32	2.21	2.57	1.70	1.98	**T = −5°C**
(PEG)_1000_	1.473	2.394	2.78	1.63	1.89	1.094	1.883	2.18	1.72	1.99	1.38	2.30	2.75	1.633	1.99	**T = RT 19°C**

r_m_: Single bond length of Carbon – Carbon.

r′_m_: Single bond length of Carbon –Oxygen.

r’‘_m_: Double bond length of Carbon – Carbon.

r_m_/r_1_: Geometrical factor.

The absence of √2 diameter and presence of the geometrical factor corresponds to ∼√3 and ∼2 diameter demonstrates the presence of an irregular unit cell with icosahedral symmetry (Frank unit) in solid state PEG.

***Solid state PEG with heat exchange: (PEG)200:*** The interference function *F(K)* derived from the coherent diffuse scattering of solid (PEG)_200_ at −5°C showed a similar behaviour to liquid (PEG)_200_ at 0°C. The FWHM in this curve ΔK = 2 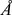 ^−1^ was the same as the solid sample (PEG)_1000_ without heat exchange at room temperature and increased value compared to ΔKliquid = 1.5 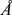 ^−1^. As suggested in the previous section this could be interpreted as a greater degree of disorder for the solid polymer. This disorder was associated to the far atomic correlation. The *W(r)* curve clearly confirmed this assumption. The reason was that beyond 10 Å there were no maxima and the slope had a value of −1. This indicated that there was not any oscillation as the behaviour was a geometrical line. The behaviour of this geometrical line also permitted the density to be calculated from the x-ray measurements as used for crystalline substances. This method of determining the density was compared to the macroscopic density determined by classical methods [6].

We next investigated the effect of temperature variation from room temperature down to −20°C at different cooling rates. Contrary to the (PEG)_1000_, in this case heat exchange was involved. The real full image of Fourier space was obtained before and after the solidification process. We observed that there was a progression in the solidification but there was only one unique image for the solid sample, which consisted of lines superimposed on coherent diffuse scattering. Therefore the change in the “physical structure” could not be considered as a solidification process as classical solidification occurred by nucleation and growth.

Our investigations have illustrated that in the case of (PEG)_200_ and (PEG)_1000_ only specific maxima from the *W(r)* could be attributed to determining the O-O bond length. The same goes for the case of liquid PEG. The maximum that corresponded to 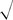
2 diameter was absent which was one of the main criteria for non-crystalline substances. Moreover, the presence of geometrical doublets of r_2_/r_1_ varied between (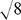/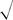 3) and 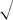
3 and r_3_/r_1_ ≈2 were also characterisations for non-crystalline substances (Table 4). Any of these geometrical models with different geometrical factors expressed the state of the sample and could be adopted for non-cystalline matter. Our results from anomalous diffractometry showed the interatomic fluctuations on the local topology. We suggest that a heap of particles cannot be only defined by a mathematical model, contrary to the pile of atoms in unit cells of periodic substances.

## Conclusions

The point of studying the atomic structure of liquid PEG by anomalous diffractometry was to determine the differences between the atomic arrangements of liquid and solid PEG.

This method provided a full real image of Fourier space of the ensemble the samples. Precise values of interatomic distances or bond lengths in atomic network were obtained by analysing these images using Fourier transformation. We conclude that the interatomic distances in liquid polymers cannot be accounted for by just assigning a fixed atomic radius of carbon atoms derived from diamond (i.e. r = 1.5433/2 Å) as has been referred to by researchers in polymer science.

We show that the atomic radius in liquid PEG is dependent on the polymerisation index (n) or the temperature of liquid. Hence, the concept of apparent size of atoms in crystalline materials is also valid (the Hume-Rothrey rule) for liquid polymers. This radius modification occurs to maintain the high stability of liquid polymers. The feature of *W(r)* and modification of the geometrical factor, obtained from experimental *W(r)* curves (r_m_/r_1_), confirm that the dense packing of Frank unit, with icosahedra symmetry, is a reasonable description of the atomic arrangement in these liquids.

The value of the geometrical factors obtained from experimental, *W(r)*, curves are consistent with each dense packing geometrical model. They show the predominant local order geometry to the determined lattice structure in periodic space. Moreover the analysis of *W(r)* curves clearly reveals the absence of O-O bond lengths at the first neighbouring distance and presence of C = C double bonds. From the absence of the O-O distance, at the first neighbour, in the atomic network of these liquids we determined the manner by which the Frank units are linked together to form a pentagonal Hume-Rothrey chain of C-C, C = C and C-O. We have shown that in the atomic structure of this binary liquid [AB] that atom [B] prefers atom [A] rather than [B], as its nearest neighbour. The analysis of the Fourier space image of these liquids indicated a shorter Pauling single bond length, C-C with a bond order n = 1. The evidence of the Pauling double bond C = C with bond order n = 2, including the resonance effect, directly showed the correlation of geometrical structure determination with the electronic structure. Moreover, we confirm experimentally the “Pauling hypothesis” for non-crystalline substances. That is: the presence of C = C double bonds and their resonance energy impose the extra stability for these liquids.

The singularity of this atomic arrangement, with the presence of pentagonal chains and chemical ordering of these liquid structures, prevent the occurrence of phase transitions from liquid PEG to crystalline solids. Under these conditions, liquid PEG needs to deplete the extra volume from the Frank unit to form crystalline seeds (clusters), whereby the topology of lattice points are fixed Cartesian coordinates. This is contrary to the dense packing of the Frank unit with the icosahedra symmetry. Consequently each atom in this atomic arrangement is free to choose two positions: either in the base (trio) or at the apex of any regular tetrahedron in the atomic network. This position of uncertainty ensures the proper filling of space by changing the geometrical factor. The geometrical factor can be measured by radial atomic distribution functions.

The variation of the geometrical factor can be interpreted as an arranged topology where the concept of disorder, can be described mathematically (geometry). The site of atoms at the apex position is at the “*ultimate position*” in the Frank unit with an icosahedral symmetry. This uncertainty provides a distorted tetrahedron; hence the degree of icosohedrality of a regular tetrahedron needs to be changed in order to fill the space. Here the variation of the degree of icosohedrality has been shown by the experimental data, obtained from anomalous diffractometry, for different samples under different conditions. Any contact between one atom and another atom may result in an uncertain deformation; our results strongly support the idea of “positional uncertainty”. This is contrary to classical mechanics where the atomic position is well defined and the probability of determining its position is introduced for finding the average position. Until now this convenient tool has been utilised for describing the morphology of polymer chains.

For filling the space the geometrical factor changes resulting in liquid PEG to remain as a perpetual super-cooled liquid. We observed this phenomenon by freezing the liquids at different temperatures, cooling rates and by rapid quenching in the liquid nitrogen. Under these conditions when the liquid transforms to a solid state the behaviour of the full real image of Fourier space does not change and always consists of lines superimposed on the coherent diffuse scattering. Even with the increase of the molecular weight to M = 1000 the image of the Fourier space remains similar. This image is characteristic of what is known as an intermediary structure.

The conclusion of this original investigation is that there is an intermediate ordered structure, which has been characterised by determining the real full image of Fourier space. This indicates the presence of a prominent state of matter, which is defined by a regular unit cell with a five-fold symmetry. These structural atomic studies illustrate an original way for understanding mechanical properties, such as the strength of materials. They contribute to a more detailed understanding of the properties of polymers than the traditional studies of the degree of crystallinity, which are derived from the concept of the two-phase model.
